# Genomic–transcriptomic evolution in lung cancer and metastasis

**DOI:** 10.1038/s41586-023-05706-4

**Published:** 2023-04-12

**Authors:** Carlos Martínez-Ruiz, James R. M. Black, Clare Puttick, Mark S. Hill, Jonas Demeulemeester, Elizabeth Larose Cadieux, Kerstin Thol, Thomas P. Jones, Selvaraju Veeriah, Cristina Naceur-Lombardelli, Antonia Toncheva, Paulina Prymas, Andrew Rowan, Sophia Ward, Laura Cubitt, Foteini Athanasopoulou, Oriol Pich, Takahiro Karasaki, David A. Moore, Roberto Salgado, Emma Colliver, Carla Castignani, Michelle Dietzen, Ariana Huebner, Maise Al Bakir, Miljana Tanić, Thomas B. K. Watkins, Emilia L. Lim, Ali M. Al-Rashed, Danny Lang, James Clements, Daniel E. Cook, Rachel Rosenthal, Gareth A. Wilson, Alexander M. Frankell, Sophie de Carné Trécesson, Philip East, Nnennaya Kanu, Kevin Litchfield, Nicolai J. Birkbak, Allan Hackshaw, Stephan Beck, Peter Van Loo, Mariam Jamal-Hanjani, TRACERx Consortium, Carlos Martínez-Ruiz, James R. M. Black, Clare Puttick, Mark S. Hill, Jonas Demeulemeester, Elizabeth Larose Cadieux, Kerstin Thol, Selvaraju Veeriah, Cristina Naceur-Lombardelli, Antonia Toncheva, Paulina Prymas, Andrew Rowan, Sophia Ward, Foteini Athanasopoulou, Oriol Pich, Takahiro Karasaki, David A. Moore, Roberto Salgado, Emma Colliver, Carla Castignani, Michelle Dietzen, Ariana Huebner, Miljana Tanić, Thomas B. K. Watkins, Rachel Rosenthal, Gareth A. Wilson, Nnennaya Kanu, Allan Hackshaw, Stephan Beck, Mariam Jamal-Hanjani, Nicholas McGranahan, Charles Swanton, Maise Al Bakir, Emilia L. Lim, Alexander M. Frankell, Kevin Litchfield, Nicolai J. Birkbak, Peter Van Loo, Jason F. Lester, Amrita Bajaj, Apostolos Nakas, Azmina Sodha-Ramdeen, Keng Ang, Mohamad Tufail, Mohammed Fiyaz Chowdhry, Molly Scotland, Rebecca Boyles, Sridhar Rathinam, Claire Wilson, Domenic Marrone, Sean Dulloo, Dean A. Fennell, Gurdeep Matharu, Jacqui A. Shaw, Joan Riley, Lindsay Primrose, Ekaterini Boleti, Heather Cheyne, Mohammed Khalil, Shirley Richardson, Tracey Cruickshank, Gillian Price, Keith M. Kerr, Sarah Benafif, Kayleigh Gilbert, Babu Naidu, Akshay J. Patel, Aya Osman, Christer Lacson, Gerald Langman, Helen Shackleford, Madava Djearaman, Salma Kadiri, Gary Middleton, Angela Leek, Jack Davies Hodgkinson, Nicola Totten, Angeles Montero, Elaine Smith, Eustace Fontaine, Felice Granato, Helen Doran, Juliette Novasio, Kendadai Rammohan, Leena Joseph, Paul Bishop, Rajesh Shah, Stuart Moss, Vijay Joshi, Philip Crosbie, Fabio Gomes, Kate Brown, Mathew Carter, Anshuman Chaturvedi, Lynsey Priest, Pedro Oliveira, Colin R. Lindsay, Fiona H. Blackhall, Matthew G. Krebs, Yvonne Summers, Alexandra Clipson, Jonathan Tugwood, Alastair Kerr, Dominic G. Rothwell, Elaine Kilgour, Caroline Dive, Hugo J. W. L. Aerts, Roland F. Schwarz, Tom L. Kaufmann, Zoltan Szallasi, Judit Kisistok, Mateo Sokac, Miklos Diossy, Abigail Bunkum, Aengus Stewart, Alastair Magness, Angeliki Karamani, Benny Chain, Brittany B. Campbell, Chris Bailey, Christopher Abbosh, Clare E. Weeden, Claudia Lee, Corentin Richard, Crispin T. Hiley, David R. Pearce, Despoina Karagianni, Dhruva Biswas, Dina Levi, Elena Hoxha, Emma Nye, Eva Grönroos, Felip Gálvez-Cancino, Francisco Gimeno-Valiente, George Kassiotis, Georgia Stavrou, Gerasimos Mastrokalos, Haoran Zhai, Helen L. Lowe, Ignacio Garcia Matos, Jacki Goldman, James L. Reading, Javier Herrero, Jayant K. Rane, Jerome Nicod, Jie Min Lam, John A. Hartley, Karl S. Peggs, Katey S. S. Enfield, Kayalvizhi Selvaraju, Kevin W. Ng, Kezhong Chen, Krijn Dijkstra, Kristiana Grigoriadis, Krupa Thakkar, Leah Ensell, Mansi Shah, Marcos Vasquez Duran, Maria Litovchenko, Mariana Werner Sunderland, Michelle Leung, Mickael Escudero, Mihaela Angelova, Monica Sivakumar, Olga Chervova, Olivia Lucas, Othman Al-Sawaf, Philip Hobson, Piotr Pawlik, Richard Kevin Stone, Robert Bentham, Robert E. Hynds, Roberto Vendramin, Sadegh Saghafinia, Saioa López, Samuel Gamble, Seng Kuong Anakin Ung, Sergio A. Quezada, Sharon Vanloo, Simone Zaccaria, Sonya Hessey, Stefan Boeing, Supreet Kaur Bola, Tamara Denner, Teresa Marafioti, Thanos P. Mourikis, Victoria Spanswick, Vittorio Barbè, Wei-Ting Lu, William Hill, Wing Kin Liu, Yin Wu, Yutaka Naito, Zoe Ramsden, Catarina Veiga, Gary Royle, Charles-Antoine Collins-Fekete, Francesco Fraioli, Paul Ashford, Tristan Clark, Martin D. Forster, Siow Ming Lee, Elaine Borg, Mary Falzon, Dionysis Papadatos-Pastos, James Wilson, Tanya Ahmad, Alexander James Procter, Asia Ahmed, Magali N. Taylor, Arjun Nair, David Lawrence, Davide Patrini, Neal Navani, Ricky M. Thakrar, Sam M. Janes, Emilie Martinoni Hoogenboom, Fleur Monk, James W. Holding, Junaid Choudhary, Kunal Bhakhri, Marco Scarci, Martin Hayward, Nikolaos Panagiotopoulos, Pat Gorman, Reena Khiroya, Robert C. M. Stephens, Yien Ning Sophia Wong, Steve Bandula, Abigail Sharp, Sean Smith, Nicole Gower, Harjot Kaur Dhanda, Kitty Chan, Camilla Pilotti, Rachel Leslie, Anca Grapa, Hanyun Zhang, Khalid AbdulJabbar, Xiaoxi Pan, Yinyin Yuan, David Chuter, Mairead MacKenzie, Serena Chee, Aiman Alzetani, Judith Cave, Lydia Scarlett, Jennifer Richards, Papawadee Ingram, Silvia Austin, Eric Lim, Paulo De Sousa, Simon Jordan, Alexandra Rice, Hilgardt Raubenheimer, Harshil Bhayani, Lyn Ambrose, Anand Devaraj, Hema Chavan, Sofina Begum, Silviu I. Buderi, Daniel Kaniu, Mpho Malima, Sarah Booth, Andrew G. Nicholson, Nadia Fernandes, Pratibha Shah, Chiara Proli, Madeleine Hewish, Sarah Danson, Michael J. Shackcloth, Lily Robinson, Peter Russell, Kevin G. Blyth, Craig Dick, John Le Quesne, Alan Kirk, Mo Asif, Rocco Bilancia, Nikos Kostoulas, Mathew Thomas, Charles Swanton, Nicholas McGranahan

**Affiliations:** 1 Cancer Research UK Lung Cancer Centre of Excellence, University College London Cancer Institute, London, UK; 2 Cancer Genome Evolution Research Group, Cancer Research UK Lung Cancer Centre of Excellence, University College London Cancer Institute, London, UK; 3 Cancer Evolution and Genome Instability Laboratory, The Francis Crick Institute and University College London Cancer Institute, London, UK; 4 Cancer Genomics Laboratory, The Francis Crick Institute, London, UK; 5 Integrative Cancer Genomics Laboratory, Department of Oncology, KU Leuven, Leuven, Belgium; 6 VIB–KU Leuven Center for Cancer Biology, Leuven, Belgium; 7 Medical Genomics, University College London Cancer Institute, London, UK; 8 Advanced Sequencing Facility, The Francis Crick Institute, London, UK; 9 Cancer Metastasis Laboratory, University College London Cancer Institute, London, UK; 10 Department of Cellular Pathology, University College London Hospitals, London, UK; 11 Department of Pathology, ZAS Hospitals, Antwerp, Belgium; 12 Division of Research, Peter MacCallum Cancer Centre, Melbourne, Victoria Australia; 13 Experimental Oncology, Institute for Oncology and Radiology of Serbia, Belgrade, Serbia; 14 Centre for Nephrology, Division of Medicine, University College London, London, UK; 15 Scientific Computing STP, Francis Crick Institute, London, UK; 16 Oncogene Biology Laboratory, The Francis Crick Institute, London, UK; 17 Bioinformatics and Biostatistics, The Francis Crick Institute, London, UK; 18 Tumour Immunogenomics and Immunosurveillance Laboratory, University College London Cancer Institute, London, UK; 19 Department of Molecular Medicine, Aarhus University Hospital, Aarhus, Denmark; 20 Department of Clinical Medicine, Aarhus University, Aarhus, Denmark; 21 Bioinformatics Research Centre, Aarhus University, Aarhus, Denmark; 22 Cancer Research UK & UCL Cancer Trials Centre, London, UK; 23 Department of Genetics, The University of Texas MD Anderson Cancer Center, Houston, TX USA; 24 Department of Genomic Medicine, The University of Texas MD Anderson Cancer Center, Houston, TX USA; 25 Department of Medical Oncology, University College London Hospitals, London, UK; 26 Singleton Hospital, Swansea Bay University Health Board, Swansea, UK; 27 University Hospitals of Leicester NHS Trust, Leicester, UK; 28 University of Leicester, Leicester, UK; 29 Cancer Research Centre, University of Leicester, Leicester, UK; 30 Royal Free Hospital, Royal Free London NHS Foundation Trust, London, UK; 31 Aberdeen Royal Infirmary NHS Grampian, Aberdeen, UK; 32 Department of Medical Oncology, Aberdeen Royal Infirmary NHS Grampian, Aberdeen, UK; 33 University of Aberdeen, Aberdeen, UK; 34 Department of Pathology, Aberdeen Royal Infirmary NHS Grampian, Aberdeen, UK; 35 The Whittington Hospital NHS Trust, London, UK; 36 Birmingham Acute Care Research Group, Institute of Inflammation and Ageing, University of Birmingham, Birmingham, UK; 37 University Hospital Birmingham NHS Foundation Trust, Birmingham, UK; 38 Institute of Immunology and Immunotherapy, University of Birmingham, Birmingham, UK; 39 Manchester Cancer Research Centre Biobank, Manchester, UK; 40 Wythenshawe Hospital, Manchester University NHS Foundation Trust, Wythenshawe, UK; 41 Division of Infection, Immunity and Respiratory Medicine, University of Manchester, Manchester, UK; 42 Cancer Research UK Lung Cancer Centre of Excellence, University of Manchester, Manchester, UK; 43 The Christie NHS Foundation Trust, Manchester, UK; 44 Division of Cancer Sciences, The University of Manchester and The Christie NHS Foundation Trust, Manchester, UK; 45 Cancer Research UK Manchester Institute Cancer Biomarker Centre, University of Manchester, Manchester, UK; 46 Artificial Intelligence in Medicine (AIM) Program, Massachusetts General Brigham, Harvard Medical School, Boston, MA USA; 47 Department of Radiation Oncology, Brigham and Women’s Hospital, Dana-Farber Cancer Institute, Harvard Medical School, Boston, MA USA; 48 Radiology and Nuclear Medicine, CARIM & GROW, Maastricht University, Maastricht, the Netherlands; 49 Institute for Computational Cancer Biology, Center for Integrated Oncology (CIO), Cancer Research Center Cologne Essen (CCCE), Faculty of Medicine and University Hospital Cologne, University of Cologne, Cologne, Germany; 50 Berlin Institute for the Foundations of Learning and Data (BIFOLD), Berlin, Germany; 51 Berlin Institute for Medical Systems Biology, Max Delbrück Center for Molecular Medicine in the Helmholtz Association (MDC), Berlin, Germany; 52 Danish Cancer Society Research Center, Copenhagen, Denmark; 53 Computational Health Informatics Program, Boston Children’s Hospital, Boston, MA USA; 54 Department of Bioinformatics, Semmelweis University, Budapest, Hungary; 55 Department of Physics of Complex Systems, ELTE Eötvös Loránd University, Budapest, Hungary; 56 Computational Cancer Genomics Research Group, University College London Cancer Institute, London, UK; 57 The Francis Crick Institute, London, UK; 58 University College London Cancer Institute, London, UK; 59 Bill Lyons Informatics Centre, University College London Cancer Institute, London, UK; 60 Experimental Histopathology, The Francis Crick Institute, London, UK; 61 Retroviral Immunology Group, The Francis Crick Institute, London, UK; 62 Department of Infectious Disease, Faculty of Medicine, Imperial College London, London, UK; 63 Department of Haematology, University College London Hospitals, London, UK; 64 Cancer Immunology Unit, Research Department of Haematology, University College London Cancer Institute, London, UK; 65 Department of Molecular Oncology and Immunology, the Netherlands Cancer Institute, Amsterdam, The Netherlands; 66 Oncode Institute, Utrecht, The Netherlands; 67 Immune Regulation and Tumour Immunotherapy Group, Cancer Immunology Unit, Research Department of Haematology, University College London Cancer Institute, London, UK; 68 Centre for Medical Image Computing, Department of Medical Physics and Biomedical Engineering, University College London, London, UK; 69 Department of Medical Physics and Bioengineering, University College London Cancer Institute, London, UK; 70 Department of Medical Physics and Biomedical Engineering, University College London, London, UK; 71 Institute of Nuclear Medicine, Division of Medicine, University College London, London, UK; 72 Institute of Structural and Molecular Biology, University College London, London, UK; 73 University College London, London, UK; 74 Department of Radiology, University College London Hospitals, London, UK; 75 UCL Respiratory, Department of Medicine, University College London, London, UK; 76 Department of Thoracic Surgery, University College London Hospital NHS Trust, London, UK; 77 Lungs for Living Research Centre, UCL Respiratory, University College London, London, UK; 78 Department of Thoracic Medicine, University College London Hospitals, London, UK; 79 University College London Hospitals, London, UK; 80 The Institute of Cancer Research, London, UK; 81 The University of Texas MD Anderson Cancer Center, Houston, TX USA; 82 Independent Cancer Patients’ Voice, London, UK; 83 University Hospital Southampton NHS Foundation Trust, Southampton, UK; 84 Department of Oncology, University Hospital Southampton NHS Foundation Trust, Southampton, UK; 85 Academic Division of Thoracic Surgery, Imperial College London, London, UK; 86 Royal Brompton and Harefield Hospitals, Guy’s and St Thomas’ NHS Foundation Trust, London, UK; 87 Department of Histopathology, Royal Brompton and Harefield Hospitals, Guy’s and St Thomas’ NHS Foundation Trust, London, UK; 88 National Heart and Lung Institute, Imperial College London, London, UK; 89 Royal Surrey Hospital, Royal Surrey Hospitals NHS Foundation Trust, Guilford, UK; 90 University of Surrey, Guilford, UK; 91 Sheffield Teaching Hospitals NHS Foundation Trust, Sheffield, UK; 92 Liverpool Heart and Chest Hospital, Liverpool, UK; 93 Princess Alexandra Hospital, The Princess Alexandra Hospital NHS Trust, Harlow, UK; 94 School of Cancer Sciences, University of Glasgow, Glasgow, UK; 95 Cancer Research UK Beatson Institute, Glasgow, UK; 96 Queen Elizabeth University Hospital, Glasgow, UK; 97 NHS Greater Glasgow and Clyde, Glasgow, UK; 98 NHS Greater Glasgow and Clyde Pathology Department, Queen Elizabeth University Hospital, Glasgow, UK; 99 Golden Jubilee National Hospital, Clydebank, UK

**Keywords:** Cancer genomics, Non-small-cell lung cancer, Transcriptomics, Tumour heterogeneity, Epigenomics

## Abstract

Intratumour heterogeneity (ITH) fuels lung cancer evolution, which leads to immune evasion and resistance to therapy^1^. Here, using paired whole-exome and RNA sequencing data, we investigate intratumour transcriptomic diversity in 354 non-small cell lung cancer tumours from 347 out of the first 421 patients prospectively recruited into the TRACERx study^2,3^. Analyses of 947 tumour regions, representing both primary and metastatic disease, alongside 96 tumour-adjacent normal tissue samples implicate the transcriptome as a major source of phenotypic variation. Gene expression levels and ITH relate to patterns of positive and negative selection during tumour evolution. We observe frequent copy number-independent allele-specific expression that is linked to epigenomic dysfunction. Allele-specific expression can also result in genomic–transcriptomic parallel evolution, which converges on cancer gene disruption. We extract signatures of RNA single-base substitutions and link their aetiology to the activity of the RNA-editing enzymes ADAR and APOBEC3A, thereby revealing otherwise undetected ongoing APOBEC activity in tumours. Characterizing the transcriptomes of primary–metastatic tumour pairs, we combine multiple machine-learning approaches that leverage genomic and transcriptomic variables to link metastasis-seeding potential to the evolutionary context of mutations and increased proliferation within primary tumour regions. These results highlight the interplay between the genome and transcriptome in influencing ITH, lung cancer evolution and metastasis.

## Abstract (web summary)

Computational and machine-learning approaches that integrate genomic and transcriptomic variation from paired primary and metastatic non-small cell lung cancer samples from the TRACERx cohort reveal the role of transcriptional events in tumour evolution.

## Main

An understanding of the causes of cancer cell-to-cell variation is essential to understand tumour evolution. Recent work has emphasized that much of this variation is transcriptomic, arising from diverse mechanisms that relate to, or are independent of, genomic variation^4^. In mouse models of non-small cell lung cancer (NSCLC), transcriptomic plasticity has been shown to underpin ITH^5^. While genomic variation reflects the relics of past somatic events acquired during the evolutionary history of a tumour, transcriptomic variation may provide an accurate approximation of the phenotypic state of a tumour at the time of sampling^1^. To date, most studies of tumour evolution in humans have focused on the impact of genomic alterations on cancer. Transcriptomic studies that leverage bulk tumour RNA sequencing (RNA-seq) data tend to focus on the amplitude of gene expression in a single biopsy taken at a single time point. This approach might fail to capture poorly understood transcriptomic processes, including allele-specific expression (ASE) and RNA editing that can exert important effects on cancer evolution^1,4^.

Here we leverage multiregion sequencing data from patients recruited into the TRACERx study^2^ to better understand the impact of multiple transcriptomic features and their interplay with genomic and phenotypic diversity in NSCLC evolution at different spatial and temporal scales.

## Cohort overview

We analysed matched RNA-seq and whole-exome sequencing data from 347 patients recruited into the prospective study TRACERx (TRACERx 421 cohort). Samples from the cohort comprised 947 tumour regions from 354 NSCLC tumours (6 patients harboured multiple primaries at diagnosis), as well as 96 tumour-adjacent normal lung tissue regions (see the consolidated standards of reporting trials (CONSORT) diagram in Supplementary Information)^6,7^. Of these patients, 344 had 886 primary tumour regions, 21 also had 29 metastatic lymph node (LN) regions sampled at surgical resection of the primary tumour and 24 patients had 30 metastatic tumour regions sampled at relapse or progression. In total, 168 primary tumour regions and 4 LN regions from 64 patients in this cohort were previously described in the TRACERx 100 cohort^8^. The cohort of paired primary–metastatic regions analysed here (and reported in a companion paper^6^) comprises 61 metastatic regions including LN regions and intrapulmonary metastases resected at surgery (henceforth termed primary LN/satellite lesions) and LN and metastatic regions at recurrence or progression.

## Expression diversity in NSCLC evolution

**Extended Data Fig. 1:**
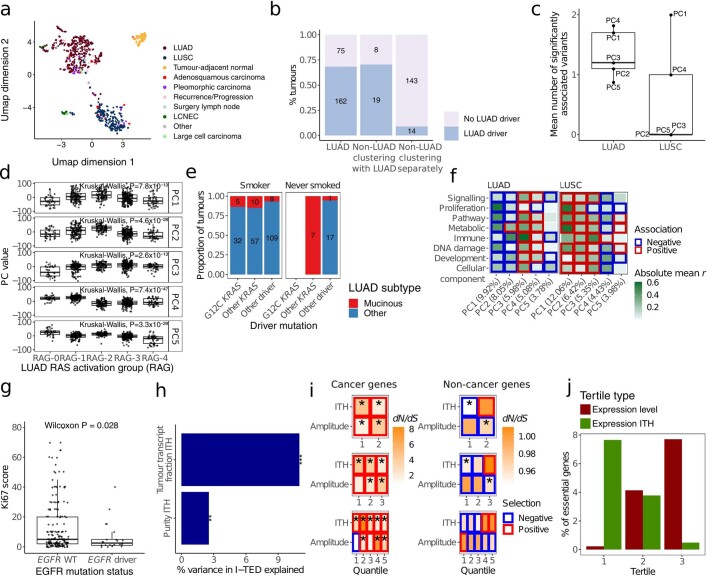
Patterns of expression diversity in the TRACERx cohort. **a**. Uniform manifold approximation and projection (UMAP) showing the distribution of each primary tumour region in the cohort based on gene expression. n = 914 tumour regions collected at surgical resection from 352 primary tumours, n = 33 recurrence/relapse samples from 24 tumours and n = 96 paired normal samples from 96 tumours. LUAD: Lung adenocarcinoma; LUSC: Lung squamous cell carcinoma; LCNEC: Large cell neuroendocrine carcinoma. **b**. Percentage of tumours with and without ‘LUAD drivers’ (driver mutations enriched in LUADs) in LUAD, non-LUADs clustering with LUADs in the UMAP and non-LUADs clustering apart from LUADs. Number of tumours within each category is annotated. **c**. Mean number of variables significantly associated with each principal component (PC) of gene expression after randomly sub-sampling the number of LUAD regions to match that of LUSC regions (n = 303) for 50 iterations. LUAD subtypes were not included in this comparison to ensure an equal number of variables between LUAD and LUSC. **d**. PC associations with each of the different RAS activation groups (RAG) developed by East and colleagues^11^. PC activity different significantly between RAGs. Analysis based on 480 tumour regions collected at surgical resection from 190 LUAD tumours where RAG could be estimated. **e**. Proportion of LUAD tumours in smokers (comprising current and ex-smokers) and never smokers, split by LUAD subtype, with either G12C KRAS driver mutations, non-G12C KRAS driver mutations or driver mutations in other genes. Numbers annotated indicate the number of tumours per category. **f**. Pearson’s *r* between each PC and functional groups comprising the fifty MSigDb Hallmark gene sets^14^. Pearson’s *r* values were averaged within the functional group to which each hallmark was assigned^14^ across LUAD, n = 480 tumour regions from 190 tumours; and LUSC, n = 303 tumour regions from 119 tumours. The colour of the border around each square indicates the direction of the association between each covariate and PC for significant (FDR<0.05) associations. Significance was determined through a mixed effects linear model using purity as a fixed covariate and tumour as a random variable; P values were calculated by hallmark and combined within MSigDB functional group using the harmonic mean. **g**. Immuno-histochemical staining for Ki67 proliferation marker in LUAD tumours with and without *EGFR* driver mutations. Only the 196 LUAD tumours within which Ki67 was measured are displayed. Significance was calculated through a two-sided unpaired Wilcoxon test. WT: Wild type. **h**. Percentage of variance in Intra-Tumour Expression Distance (I-TED) that was explained by intra-tumour variance in tumour transcript fraction and intra-tumour variance in tumour purity, in a linear regression. Analysis based on 258 tumours with at least two primary tumour regions, and purity and tumour transcript fraction estimates. ***:P value = 5.03 × 10^−8^; **:P value = 0.007. **i**. dN/dS in non-cancer and cancer genes for different quantiles of ITH or expression amplitude. Asterisks indicate significance whereby the 95% confidence interval of the dN/dS estimate did not overlap 1 signalling either negative (blue square) or positive (red square) selection. Broadly, lower quantiles of ITH tended towards negative selection in non-cancer genes, whereas the opposite was true for cancer genes. Results based on bootstrapping from the total number of tumour samples resected at surgery of the primary tumour from tumours with more than one sample at that time point, 845 regions from 285 tumours. **j**. Percentage of all essential genes from the Project Achilles list^18^ (n = 604) in lung cancer for tertiles of expression ITH or amplitude. All box plots in this figure represent lower quartile, median and upper quartile, whiskers represent lower/higher bound +/− 1.5 x interquartile range.

We first examined patterns of gene expression across tumour samples. A uniform manifold approximation and projection (UMAP) analysis (Extended Data Fig. 1a) based on gene expression across the cohort revealed that samples clustered in three main groups dominated by lung adenocarcinomas (LUADs), lung squamous cell carcinomas (LUSCs) and tumour-adjacent normal lung tissue. Notably, 27 out of 184 non-LUAD tumours, defined by central pathological review, clustered with LUADs. These tumours, which included four LUSCs, were 23 times more likely to harbour a LUAD-specific driver mutation (Methods) than other non-LUADs (*P* = 2.7 × 10^−11^, Fisher’s exact test; Extended Data Fig. 1b). Although not classified as LUADs, 67% of these tumours (18 out of 27) were positive for common LUAD immunohistochemical staining markers such as TTF-1 or exhibited LUAD morphology (Extended Data Table 1). This enrichment for LUAD driver mutations among non-LUAD NSCLC tumours that cluster with LUADs suggests that phenotypically, this subset of tumours may be similar to LUADs. This result is also consistent with some such tumours harbouring an adenocarcinomatous component^9^ and with other reports of LUAD drivers in non-LUAD tumours^10^.

**Fig. 1:**
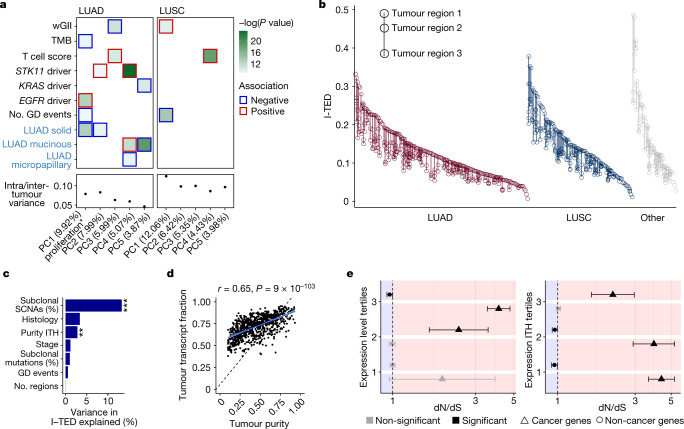
Expression diversity in the TRACERx 421 cohort. **a**, Relationship between PCs of transcriptomic diversity and genomic (black labels) and clinical (blue labels) variables. Displayed are the top PCs within LUADs (*n* = 480 regions from 190 tumours) and LUSCs (*n* = 303 regions from 119 tumours) that together explain at least 30% of the total variance, alongside their median ratio of heterogeneity (intratumour heterogeneity of PC activity divided by intertumour heterogeneity of PC activity). The colour of the border around each square indicates the direction of the association between each covariate and PC. In total, 39 variables were tested (Methods). Significance was determined using a mixed-effects linear model with purity as a fixed covariate and tumour as a random variable. Only features significant (*P* < 0.05) after FDR correction with at least one PC are displayed. *PC1 in LUAD was strongly negatively associated with the expression of hallmark gene sets related to proliferation (Extended Data Fig. 1f, Methods). GD, genome doubling; TMB, tumour mutational burden; wGII, weighted genome instability index. **b**, I-TED, calculated as the mean normalized gene expression correlation distance for a given region paired with every other region from the same tumour, displayed by histology. **c**, Proportion of variance in I-TED explained by selected genomic and clinical features from a linear model using 260 tumours with at least 2 primary tumour regions, and purity and genome instability estimates. Histological types represented by only a single tumour were excluded to ensure a sufficiently large sample size to estimate the effect of histology. **P = 0.003, ***P = 5.15 × 10^−10^. **d**, ASCAT-derived tumour purity and RNA estimate of the tumour transcripts fraction. Each dot represents one tumour region. A modified version of ASCAT^50^ was used to estimate the proportion of tumour and non-tumour cells within an admixed sequencing sample. **e**, dN/dS, inferring positive and negative selection of truncating somatic mutations, for cancer genes and non-cancer genes, by tertiles of median gene expression across the cohort (left) and by tertiles of gene expression ITH across the cohort (right). Dots represent the estimated dN/dS and the error bars represent the 95% confidence intervals calculated using the genesetdnds function in R from the package dNdScv. A dN/dS estimate is considered significant if the 95% confidence intervals do not overlap 1. Expression level tertiles contained 76, 24 and 9 cancer genes, and 4,856, 5,100 and 5,166 non-cancer genes, for tertiles 3, 2 and 1, respectively. Expression ITH tertiles contained 54, 24 and 31 cancer genes and 4,994, 5,082 and 5,046 non-cancer genes, for tertiles 3, 2 and 1, respectively. Median expression levels and expression ITH were based on the total number of tumour samples collected at surgical resection from tumours with more than one sample at that time point (*n* = 845 regions from 283 tumours).

Next, to establish determinants of intertumour and intratumour transcriptomic diversity, we performed independent principal component analyses (PCAs) within the two major NSCLC histologies (LUAD and LUSC) and related these to 39 underlying genomic and clinico-pathological variables (Fig. 1a; see Methods for the rationale of feature selection). Principal components (PCs) were more frequently significantly correlated with genomic variables in LUAD than in LUSC. This trend persisted when LUADs were downsampled to account for differences in the sample size (Extended Data Fig. 1c). PCs exhibited lower relative ITH in LUADs compared to LUSCs; that is, the ratio of intratumour to intertumour heterogeneity of the PC amplitude was lower within LUADs (Fig. 1a). Taken together, these results are suggestive of more deterministic genomic–transcriptomic relationships within LUADs than LUSCs. Furthermore, LUAD PC activity correlated with orthogonal signatures that quantify RAS pathway activation^11^, which highlights that PCs might represent transcriptional programmes that are preserved across datasets (Extended Data Fig. 1d).

In LUADs, this analysis further revealed two relationships consistent with mutual exclusivity, with separate features showing significant and opposing correlations with a given PC. First, PC5 was positively associated with predicted driver mutations in *KRAS* and invasive mucinous adenocarcinomas (IMAs). IMAs were enriched in tumours harbouring non-G12C *KRAS* predicted driver mutations (*P* = 0.003, *χ*^2^ test; Extended Data Fig. 1e), which were less likely to be associated with a history of smoking^12^. This result provides transcriptomic context to previous work suggesting that IMAs are more common in never-smokers^13^. Second, PC1 was strongly negatively correlated with MSigDB Hallmark gene sets related to proliferation^14^ (Extended Data Fig. 1f), yet positively associated with activating mutations in *EGFR* (linear mixed-effect, model false discovery rate (FDR) = 0.0008). In keeping with this, *EGFR* driver mutations were associated with low Ki-67 levels (*P* = 0.028, Wilcoxon test; Extended Data Fig. 1g). This finding suggests that the phenotype of *EGFR* mutant LUADs is one of reduced proliferation compared with *EGFR* wild-type LUADs.

To further assess transcriptomic ITH independently from the number of tumour regions sampled, we developed the intratumour expression distance (I-TED) metric, which is calculated as the mean normalized gene expression correlation distance for a given region paired with every other region from the same tumour (Methods and Fig. 1b). A high I-TED value reflects high expression ITH. Hierarchical clustering of all samples based on the gene expression correlation distance revealed that tumour regions from a given patient tended to cluster together (in 231 out of 280 multiregion primary tumours, all regions within a given tumour clustered together). Within the 49 tumours for which constituent regions did not all cluster, those regions clustering apart harboured increased weighted genome instability index scores (*P* = 0.002, linear regression, 104 regions). Consistently, the fraction of the genome affected by subclonal somatic copy number alterations (SCNAs) and intratumour variation in purity were independently associated with increased I-TED values (Fig. 1c; 13.3% and 2.8% of variance explained, respectively). Conversely, I-TED was not associated with the heterogeneity of subclonal mutations nor the number of regions sampled per tumour. This result underlines the link between SCNAs and changes in gene expression.

To further evaluate the relationship between tumour purity and transcriptomic heterogeneity, we estimated the tumour transcript fraction (a ploidy-adjusted estimate of the proportion of all transcripts that were derived from the tumour) from RNA-seq reads (Methods). We observed that the tumour transcript fraction was consistently greater than the tumour purity (Fig. 1d). This result suggests that per chromosome copy, gene expression from tumour cells tends to exceed that of non-tumour cells within a bulk sample, which is in keeping with results from another study^15^. Of note, the tumour transcript fraction was a better predictor of I-TED than purity, which highlights that DNA-derived estimates of tumour diversity may not always be representative of phenotypic diversity (*P* = 5.03 × 10^−8^, linear regression; Extended Data Fig. 1h).

Next, we sought to understand whether patterns of gene expression and their heterogeneity are related to selection during tumour evolution. We measured selection within established lung cancer and non-cancer genes using the ratio between the observed number of nonsynonymous mutations per nonsynonymous site and the number synonymous mutations per synonymous site (dN/dS), calculated through the dNdScv method^16^. Genes were grouped into tertiles according to the average amplitude of their expression across the cohort (Fig. 1e). Within cancer genes, significant positive selection (implied when dN/dS with ±95% confidence intervals is >1) was most readily observed within truncating mutations in genes in the highest expression tertile. Notably, within non-cancer genes, signals of negative selection (dN/dS ± 95% confidence intervals of <1) were identified within truncating mutations in genes within the highest expression tertile only (242 truncating mutations, relative to 3,932 observed truncating mutations, were estimated to have been lost through negative selection in these genes). Similar patterns were observed when dividing the data by different expression quantiles (Extended Data Fig. 1i).

Expanding on this analysis, we next explored the relationship between the ITH of gene expression (measured as the standard deviation of normalized gene expression among all regions within a tumour) and selection in tumour evolution^17^. Cancer genes within the lowest tertile of expression ITH exhibited the strongest signals of positive selection. By contrast, within non-cancer genes of the same tertile, negative selection was identified (188 truncating mutations, relative to 3,083 observed truncating mutations, were estimated to have been lost through negative selection; Fig. 1e). Furthermore, the lowest ITH quantile and highest expression quantile were significantly enriched for NSCLC essential genes as identified in the Project Achilles study^18^ (*χ*^2^ test, *P* = 2.8 × 10^−81^; Extended Data Fig. 1j). These results are consistent with the idea that a subset of highly and homogeneously expressed non-cancer genes are conserved during somatic evolution and with the presence of weak negative selection among mutations in cancer.

## ASE in NSCLC

Next, we focused on transcriptomic diversity arising from ASE, which may result from genomic allelic imbalance (termed copy number (CN)-dependent ASE) or from unequal allelic expression per chromosome copy (CN-independent ASE).

We analysed genes that contained at least one heterozygous germline single-nucleotide polymorphism (SNP) with an RNA coverage of >8 reads (Methods). It was possible to evaluate ASE in a total of 16,378 different genes across all samples within the cohort at an average of 3,809 (s.d. ± 885) and 4,064 (s.d. ± 485) genes per tumour and normal tissue sample, respectively.

**Fig. 2:**
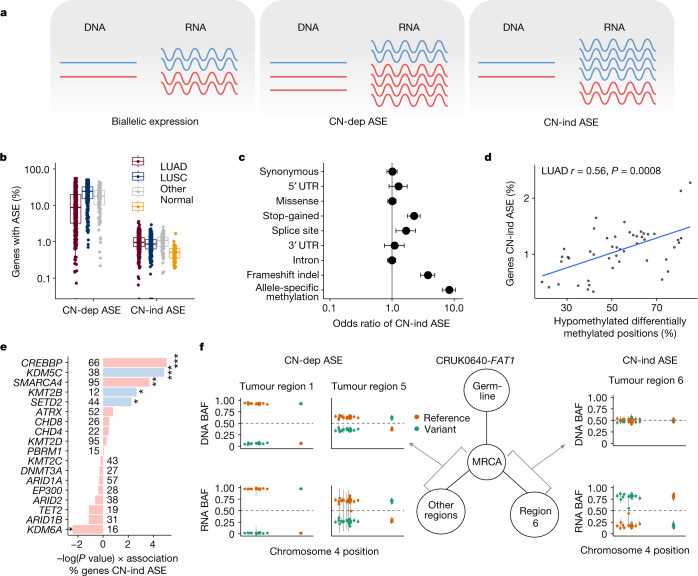
ASE in NSCLC. **a**, Schematic displaying the concepts of biallelic expression, CN-dependent ASE (CN-dep ASE) and CN-independent ASE (CN-ind ASE). **b**, Proportion of evaluable (containing an expressed SNP) genes affected by CN-dependent ASE and CN-independent ASE in tumours and normal tissue samples. LUAD, *n* = 454 regions from 144 tumours; LUSC, *n* = 293 regions from 88 tumours; Other, other subtypes, *n* = 130 regions from 38 tumours; Normal, tumour-adjacent normal lung tissue, *n* = 95. **c**, Points indicate odds ratio estimates for CN-independent ASE when somatic point mutations, or ASM (in samples for which both RRBS and RNA-seq were available) was concomitantly detected in the same gene, by type of alteration. Bars indicate 95% confidence intervals. Odds ratios for the links between CN-independent ASE and mutations and between CN-independent ASE and ASM are based on 876 primary tumour regions from 332 tumours and on 96 tumour regions from 31 tumours, respectively. **d**, Relationship in LUAD between the proportion of evaluable genes with CN-independent ASE and the ratio of differentially hypomethylated clusters of neighbouring CpGs compared to all differentially methylated genomic positions. The *P* value was calculated using a linear mixed-effects model with tumour as the random variable. **e**, Linear mixed-effects model showing the impact of driver mutations in candidate epigenetic modifier genes^22^ (mutated in more than five tumours) and tumour mutational burden on the proportion of evaluable genes with CN-independent ASE. Factors independently associated with increased CN-independent ASE in a multivariable model are coloured blue. *P < 0.05, **P < 0.01, ***P < 0.001. **f**, An example of genomic–transcriptomic mirrored subclonal allelic imbalance occurring in *FAT1* within CRUK0640. DNA and RNA B allele frequencies (BAFs) for each SNP in *FAT1* are plotted and coloured according to the reference and variant status of each allele for each region sampled within the tumour. In this instance, there is evidence of CN-dependent ASE in two regions and CN-independent ASE in one region. These events favour overexpression of different parental alleles and occur on different branches of the phylogenetic tree; a simplified version is displayed. MRCA, most recent common ancestor.

We evaluated CN-dependent and CN-independent ASE using an approach that controls for the difference in tumour purity and tumour transcript fraction of each sample (Figs. 1d and 2a and Methods)^19^. The mean percentage of evaluable genes with CN-dependent ASE in each tumour region was 17.4% (s.d. ± 12.7%), compared with 1.01% with CN-independent ASE (s.d. ± 0.47%), which partially reflects our stringent approach to calling CN-independent ASE (Fig. 2b).

**Extended Data Fig. 2:**
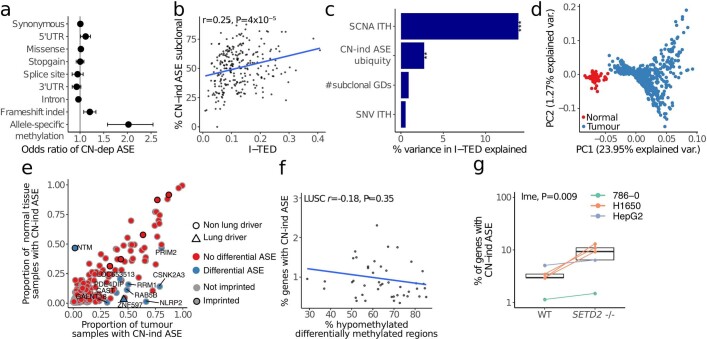
Genomic and transcriptomic links with allele-specific expression. **a**. Points indicate odds ratio estimates for copy-number dependent allele-specific expression (CN-dependent ASE) when somatic point mutations, or allele-specific methylation (where both RRBS and RNA-Seq were available) were concomitantly detected in the same gene, by type of alteration. Bars indicate 95% confidence intervals. Odds ratio for the links between CN-dependent ASE and mutations; and CN-dependent ASE and ASM are based on 876 primary tumour regions from 332 tumours, and 96 tumour regions from 31 tumours, respectively. **b**. Relationship between the proportion of CN-independent ASE in a tumour that is subclonal, being found in a subset of regions within a given tumour, and intra-tumour expression diversity. The Pearson correlation coefficient is shown (*r* = 0.25, P = 4 × 10^−5^). **c**. Percentage of variation in I-TED that was explained by single nucleotide variant (SNV), SCNA and CN-independent ASE ITH, as well as the number of subclonal whole genome duplication events (GDs) per tumour. The linear regression was based on 269 tumours where all variables could be calculated. ***:P = 2.4 × 10^−10^; **:P = 0.004. **d**. PCA of CN-independent ASE patterns in TRACERx421 tumours (n = 877 tumour regions) and normal tissue (n = 95) samples where CN-independent ASE could be estimated. Samples are coloured by tissue type. Values within parentheses on the axes indicate the proportion of variance explained by each principal component. **e**. Genes with CN-independent ASE in either tumour or normal tissue samples. Genes with an enrichment of CN-independent ASE in tumours are marked in blue, lung cancer genes are represented by triangles and imprinted genes have a black outline. Enrichment was defined as FDR < 0.05 from a Fisher’s exact test per gene. The number of regions used to calculate enrichment varied per gene between 5 and 850 (median = 164) for tumours and between 5 and 95 (median = 35) for normal tissue. **f**. Relationship in LUSC between the proportion of evaluable genes with CN-independent ASE and the ratio of differentially hypo-methylated clusters of neighbouring CpGs compared to all differentially methylated genomic positions. The Pearson correlation coefficient is shown; P value was calculated using a linear mixed-effects model with tumour as random variable (*r* = −0.18, P = 0.35). **g**. Percentage of evaluable genes affected by CN-independent ASE in wild type (WT) and *SETD2* deficient isogenic cell lines. Expression data was obtained from publicly available datasets from three separate studies in three different cell lines^23–25^: in total, data from 10 cell lines across 3 experiments (n = 6, 2 and 2). Boxes represent lower quartile, median and upper quartile. P values were calculated using a linear mixed effects model, using the study of origin of each sample as a random effect. *SETD2*-/-: inactivation of the *SETD2* gene.

ASE can result from genomic imprinting or truncating mutations that lead to nonsense-mediated decay of the mutant allele. In keeping with this, imprinted genes^20^ were significantly enriched among genes most frequently affected by CN-independent ASE (odds ratio (OR) = 71.3, *P* < 2.2 × 10^−16^) and explained 5.4% of the observed CN-independent ASE. CN-independent ASE was also enriched in genes that contained a stop-gain mutation (OR = 2.23, *P* = 1.4 × 10^−11^), an insertion or deletion leading to a frameshift (OR = 3.73, *P* < 2.2 × 10^−16^) or a splice-site mutation (OR = 1.66, *P* = 0.006) (Fig. 2c). Such mutations explained 0.7% of the total observed CN-independent ASE and had a reduced impact on CN-dependent ASE (Extended Data Fig. 2a).

ITH of CN-independent ASE, defined as the proportion of events that were detected in only a subset of the tumour regions in which it was possible to evaluate ASE, was correlated with I-TED (Pearson’s *r* = 0.25, *P* = 4 × 10^−5^; Extended Data Fig. 2b). A linear model of the determinants of I-TED revealed that the heterogeneity of SCNAs and CN-independent ASE were independent predictors of I-TED, accounting for 13.9% and 2.7% of variance, respectively (Extended Data Fig. 2c, *P* = 2.4 × 10^−10^ and *P* = 0.004, respectively). This result highlights the link between ASE and transcriptional diversity.

Next, we assessed whether patterns of CN-independent ASE varied between tumour and tumour-adjacent normal tissue samples. The lack of expressed SNPs within many genes necessitated the imputation of missing data; therefore we considered genes in which ASE was evaluable in ≥100 tumour regions across the cohort (Methods). PCA revealed that normal tissue samples were distinguishable from tumour samples, which suggests that patterns of CN-independent ASE are fundamentally different between normal tissue and tumour samples (Extended Data Fig. 2d). Gene-level analysis showed that 11 genes were subject to differential CN-independent ASE between normal and tumour tissue when controlling for repeated measures: *NTM* (more frequent CN-independent ASE in normal tissue); and *NLRP2*, *PRIM2*, *CSNK2A3*, *GALNT18*, *ZNF597*, *RAB5B*, *RRM1*, *CAST*, *PDE4DIP* and *LOC653513* (more frequent CN-independent ASE in tumours) (Extended Data Fig. 2e).

To investigate the mechanisms that underpin CN-independent ASE, we examined tumour regions (96 regions from 31 tumours) with DNA methylation data from reduced representation bisulphite sequencing (RRBS). Copy-number-aware methylation deconvolution analysis of cancers (CAMDAC)^21^ was used to estimate allele-specific methylation (ASM) rates, excluding the signal from non-cancer cells (Methods). ASM was 8.4 times more likely to occur at the promoters of genes showing CN-independent ASE than those without CN-independent ASE (*P* < 2.2 × 10^−16^, Fisher’s exact test; Fig. 2c). When global levels of methylation in a tumour region (measured as the percentage of all differentially methylated positions that comprise hypomethylated CpG loci) were compared with the proportion of evaluable genes with CN-independent ASE, a correlation was observed in LUADs but not LUSCs (Pearson’s *r* = 0.56, *P* = 0.0008, linear mixed-effects model; Fig. 2d and Extended Data Fig. 2f). In LUADs, CN-independent ASE might therefore represent a surrogate for methylation patterns.

Given the relationship between CN-independent ASE and epigenetic variation, we proposed that tumours that harbour driver mutations in epigenetic modifier genes^22^ might contain more CN-independent ASE. Consistent with this hypothesis, univariate linear regression analysis revealed that mutations within epigenetic modifier genes, in particular *CREBBP*, *KDM5C*, *SMARCA4*, *SETD2* and *KMT2B*, were associated with higher levels of CN-independent ASE. By contrast, *KDM6A* predicted driver mutations were associated with decreased CN-independent ASE (*P* < 0.05; Fig. 2e). A multivariable linear mixed-effects model, controlling for tumour mutational burden and repeated measures, confirmed that mutations in *SETD2*, *KDM5C* and *KMT2B* were independently predictive of higher levels of CN-independent ASE. To validate this observation, we explored publicly available RNA-seq data from *SETD2-*deficient isogenic human cell lines. Across H1650 (lung)^23^, 786-0 (renal)^24^ and HepG2 (liver)^25^ cell lines, we observed an increase in CN-independent ASE in *SETD2*-deficient cells compared with wild type (*P* = 0.009, linear mixed-effects model; Extended Data Fig. 2g).

Cataloguing CN-dependent and CN-independent ASE within multiregion tumours also enabled the identification of examples of parallel evolution in which genomic and transcriptomic events affecting the same gene evolve independently in different subclones within a tumour. Such events would not be detected with a genomic-only approach. We utilized haplotype phasing to explore evidence of mirrored subclonal allelic imbalance (MSAI), in which the maternal allele is gained or lost in one subclone of a tumour but the paternal allele is gained or lost in another subclone independently. We provide an example of this phenomenon in the context of allelic expression data in tumour CRUK0640 (Fig. 2f). Here the tumour suppressor gene *FAT1* contained a loss of heterozygosity with associated CN-dependent ASE in two tumour regions. However, in one other tumour region, *FAT1* did not contain a SCNA but instead showed evidence of CN-independent ASE, which might represent transcript repression favouring the expression of the parental allele subject to copy number loss in the other two regions. Phylogenetic reconstruction demonstrated that the tumour regions showing CN-dependent and CN-independent ASE were found on different branches, suggestive of parallel evolution, with convergence upon the loss of different alleles of *FAT1* through different mechanisms. This example of genomic–transcriptomic MSAI highlights that CN-independent ASE can provide an alternative source of diversity to genomic variation in an evolving cancer.

## RNA-editing diversity in NSCLC

**Fig. 3:**
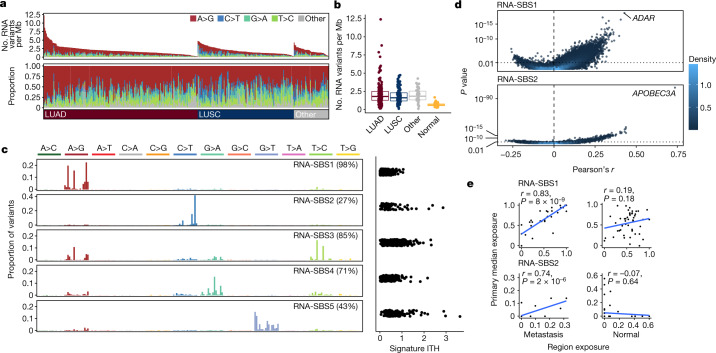
RNA-SBS signatures in NSCLC. **a**, RNA-editing overview (from top to bottom): number and type of RNA substitutions per Mb per primary tumour, tumours are sorted from left to right by histological subtype and by number of substitutions; proportion of each editing type per tumour; NSCLC histological subtype per tumour. **b**, Number of RNA substitutions detected per tumour by histological subtype of NSCLC and in normal adjacent lung tissue. LUAD, *n* = 190; LUSC, *n* = 119; Other, other subtypes, *n* = 43; Normal, tumour-adjacent normal lung tissue, *n* = 96. Boxes represent the lower quartile, median and upper quartile. **c**, Left, trinucleotide profile of each RNA-SBS signature (left). Only samples from patients with more than 20 RNA variants were considered, *n* = 333. Right, signature ITH measured as standard deviation of each signature exposure across tumour regions divided by the mean exposure of each signature across the cohort, based on 280 tumours with more than 20 RNA variants and more than one region. The percentage of tumours with signature activity in at least one primary region is indicated in parentheses. **d**, Volcano plot showing the Pearson’s *r* correlations between the number of RNA-SBS1 (top) or RNA-SBS2 (bottom) substitutions with the expression of all genes in the transcriptome. *P* values were calculated using a linear mixed-effects model, using the tumour of origin of each region as random effect. *P* values were adjusted for repeated measures. Correlations were based on 765 primary tumour regions with at least 20 RNA variants from 329 tumours. Colour indicates dot density, with light coloured points belonging to areas of high density in the plot. **e**, Correlation between the exposure of RNA-SBS signatures within tumour-adjacent normal lung tissue and their respective primary tumour regions, and metastatic tumour regions and their respective seeding regions in the primary tumour. Primary tumour exposure was calculated as the median exposure across all primary regions for the comparison with tumour-adjacent normal tissue, and across all seeding regions for the comparison with metastases. Only primary–metastasis pairs where more than 20 RNA substitutions were detected in the metastasis and primary region were used (*n* = 50 pairs for normal samples, *n* = 31 for metastases). *P* values were computed with a two-sided *t* test testing the null hypothesis that the Pearson correlation coefficient *r* = 0.

**Extended Data Fig. 3:**
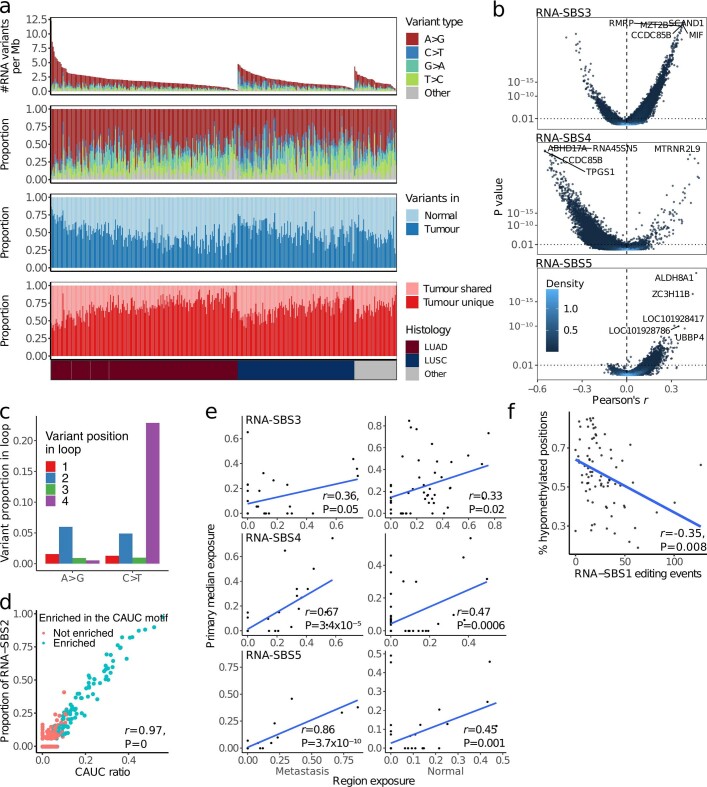
Patterns of RNA variant diversity in TRACERx. **a**. Overview of RNA substitutions in the primary tumour lung TRACERx cohort, from top to bottom: Number and type of RNA variants per megabase per tumour, tumours are sorted from left to right by histological subtype and by number of variants; Proportion of each variant type per tumour; Proportion of variants present in any of the normal samples; Proportion of tumour-specific RNA variant sites shared across at least two tumours. NSCLC histological subtype per patient. LUAD, lung adenocarcinomas, n = 190; LUSC, lung squamous cell carcinomas, n = 119; Other, other subtypes, n = 43; tumour-adjacent normal lung tissue, n = 96. **b**. Volcano plots showing Pearson correlations between the number of RNA variant signature substitutions and gene expression for all genes in the transcriptome, split by RNA single-base substitution (SBS) signature. P values were calculated using a linear mixed effects model, using tumour of origin of each region as random effect. The genes with the 5 most significant correlations with each signature are labelled. P values were adjusted for repeated measures. Correlations were based on 765 primary tumour regions with at least 20 RNA variants from 329 tumours. Colour indicates dot density, with light coloured points belonging to areas of high density in the plot. **c**. Proportion of RNA variants relative to variant type (A>G or C>T) in 4nt RNA loops. C>T substitutions were more prevalent in the 4th nucleotide of 4nt RNA hairpin loops, consistent with APOBEC RNA editing activity. **d**. Proportion of substitutions assigned to RNA-SBS2 activity compared to the proportion of RNA variants at CAT[C>T] motif sites per tumour region (CAUC ratio). Blue dots represent regions where RNA editing at these motifs was enriched (Fisher’s test P<0.05 for C>T substitutions at each site compared to C sites in a 40nt genomic region). P values were computed based on a two-sided t test testing the null hypothesis that the Pearson correlation coefficient (*r*) = 0, within 892 tumour regions and 77 tumour-adjacent normal tissue samples with at least 10 C>T variants. **e**. Pearson correlation between the exposure of RNA-SBS signatures within metastatic tumour regions and their respective seeding regions in the primary tumour (left); and tumour-adjacent normal lung tissue and their respective primary tumour regions (right). Primary tumour exposure was calculated as the median exposure across all primary regions for the comparison with normal tumour-adjacent tissue, and of all seeding regions for the comparison with metastases. Only primary-metastasis pairs where more than 20 RNA substitutions were detected in the metastasis and primary region were used (n = 50 pairs for normals, n = 31 for metastases). P values were computed based on a two-sided t test testing the null hypothesis that the Pearson correlation coefficient = 0. **f**. Pearson correlation between the activity of RNA-SBS1 and the global levels of methylation in a tumour region (measured as the percentage of all differentially methylated positions that are differentially hypomethylated clusters of neighbouring CpGs). Methylation data and sufficient RNA substitutions for signature deconvolution were available for 80 regions from 31 tumours. P values were calculated using a linear mixed effects model, using tumour of origin of each region as a random effect.

Another potential source of transcriptomic diversity is RNA editing, a post-transcriptional process characterized by changes in the nucleotide sequence of RNA molecules. We applied a stringent approach to define exonic RNA substitutions (single nucleotide changes exclusive to RNA molecules and absent in DNA) and identified 40,057 RNA substitutions across 6,019 specific sites across the cohort (mean of 1.26 RNA substitutions per Mb per tumour; Fig. 3a,b and Extended Data Fig. 3a). The majority (mean 59.7% per tumour region) were A>G substitutions, in keeping with ADAR-linked RNA editing, which deaminates adenosine to inosine, a nucleotide that is then read as guanosine by the translation machinery^26^ and sequencing platforms. Of these substitutions, 65% were present in the REDIportal database^27^ of known A>G editing events in human tissues. C>T substitutions^28^ represented 11.8% of the total substitutions detected. Of all the RNA substitutions detected, 67% were tumour specific (not present within a TRACERx panel of samples of normal tissue), and of these, 29.4% were shared between two or more tumours.

To investigate the molecular processes that underlie RNA editing in an unbiased manner, we generated RNA single-base substitution (RNA-SBS) signatures, which considered not only the mutated base but also the two adjacent bases and the strand on which the mutation occurred^29^. We detected five RNA-SBS signatures: RNA-SBS1 to RNA-SBS5 (Fig. 3c). RNA-SBS1 consisted predominantly of A>G transitions, whereas RNA-SBS2 consisted mainly of C>T transitions. RNA-SBS3 consisted mainly of A>G and T>C transitions, RNA-SBS4 of G>A transitions and RNA-SBS5 of G>T transversions. RNA-SBS1 and RNA-SBS3 were identified in most tumours (RNA-SBS1 in 98% and RNA-SBS3 in 85%). RNA-SBS1 exhibited the lowest ITH and was detected within all regions of 87.4% of multiregion tumours.

An unbiased correlation of the activity of each RNA-SBS signature with gene expression (Extended Data Fig. 3b) revealed a relationship between RNA-SBS1 and *ADAR* expression (Pearson’s *r* = 0.42, FDR = 2.4 × 10^−14^, linear mixed-effects model; Fig. 3d). The RNA 192-channel substitution spectrum (encompassing all possible trinucleotide contexts of RNA substitutions across both transcribed strands) derived when considering only those events that overlapped curated A>G sites from REDIportal was highly similar to RNA-SBS1 (cosine similarity = 0.97), consistent with the A>G activity of ADAR underpinning RNA-SBS1.

RNA-SBS2 was dominated by C>T transitions at TpC sites (67%), a motif consistent with the RNA editing activity of APOBEC3A (ref. ^30^). In keeping with this, an unbiased analysis showed that RNA-SBS2 correlated more strongly with *APOBEC3A* expression than with any other gene in the transcriptome (Pearson’s *r* = 0.73, FDR = 4.7 × 10^−108^; Fig. 3d). A multiple linear regression considering all APOBEC enzymes revealed that the expression of *APOBEC3A* was the strongest independent predictor of RNA-SBS2 activity, although *APOBEC3F* was also significant (*P* = 2.6 × 10^−57^ and *P* = 0.01 for *APOBEC3A* and *APOBEC3F*, respectively, linear mixed-effects model). Investigating the link between RNA-SBS2 and C>T enrichment at APOBEC3A-specific motifs^30,31^ further confirmed that RNA-SBS2 was strongly influenced by *APOBEC3A* expression (Extended Data Fig. 3c,d). Associations between gene expression or genomic features and the activity of the three remaining RNA-SBS signatures did not produce any obvious explanations for their aetiology.

Next we tested whether the processes that underlie RNA substitutions were also identified within paired normal tissue samples and whether they were preserved over time during cancer evolution (Fig. 3e and Extended Data Fig. 3e). For all RNA-SBS signatures, activity was correlated between metastatic regions and their paired primary tumours. However, signature activity was also preserved between tumour regions and paired normal tissue samples in the case of RNA-SBS3, RNA-SBS4 and RNA-SBS5, but not in RNA-SBS1 or RNA-SBS2. These findings suggest that the processes that underlie changes in RNA-SBS1 and RNA-SBS2 might be tumour-specific. Moreover, they might occur de novo within the regions of primary tumours that seed metastasis and persist within their metastases. By contrast, those that fuel RNA-SBS3, RNA-SBS4 and RNA-SBS5 might be influenced by germline, environmental or technical factors.

*ADAR* has previously been linked to epigenomic dysregulation^32^. Accordingly, we tested whether RNA-SBS1 activity might be influenced by epigenomic dysregulation within tumours. We observed a significant correlation between global levels of hypomethylation and RNA-SBS1 activity in tumour regions but not in paired normal tissue samples. This result highlights that these processes might be linked in NSCLC evolution (Pearson’s *r* = –0.35, *P* = 0.008, linear mixed-effects for tumour regions; *P* = 1 for normal tissue samples; Extended Data Fig. 3f).

## Multi-omic features of metastasis

**Fig. 4:**
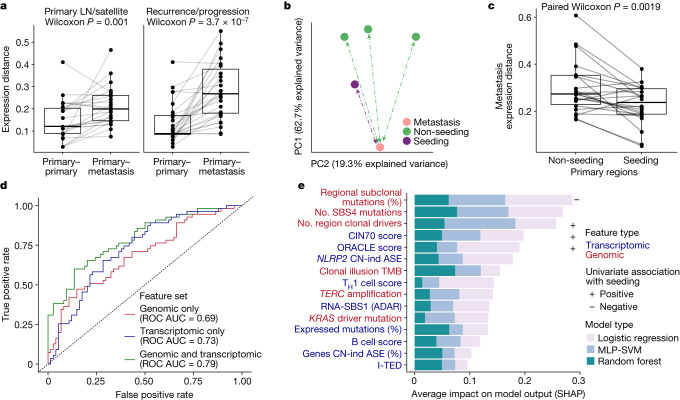
Transcriptional landscape of seeding tumour regions. **a**, Expression distance between primary regions compared to either metastatic LN regions or pulmonary nodules resected at the time of surgery (left) or metastatic regions resected at relapse within the same patient (right). Only tumours containing two or more regions with at least one metastatic region sampled are shown (*n* = 50 primary–metastasis pairs from 35 tumours). **b**, First two PCs for all available primary and metastatic tumour regions in an example tumour, CRUK0361, based on gene expression levels. The region containing the seeding clone was more proximal to the metastatic sample than other primary regions. **c**, Expression distance between metastatic samples and their paired primary samples across the cohort depending on whether the region contained a seeding clone(s). The analysis was run on 22 metastatic samples that had gene expression data for both seeding and non-seeding primary regions. **d**, ROC curves for ensemble models trained on each feature set: genomic only (red), transcriptomic only (blue), combined genomic and transcriptomic (green) and assessed against the held-out test dataset. The predictions are based on 516 primary tumour regions from 206 tumours for which seeding status could be established and for which all metrics tested could be measured (307 non-seeding regions, 209 seeding), with a 75/25% training/test dataset split. **e**, Mean Shapley additive explanations (SHAP) values (calculated across the held-out test dataset) for each feature in the combined ensemble model, capturing the importance of each feature for model prediction. Label colours indicate the feature type, genomic (red) or transcriptomic (blue), and box colours indicate the model type from which the SHAP values were extracted. The symbols at the end of the bars indicate either a significantly positive (+) or negative (–) association, with increased weight for seeding potential based on a two-sided Wilcoxon test comparing seeding to non-seeding regions. MLP-SVM, multilayer perceptron with support vector machine. All box plots in this figure represent the lower quartile, median and upper quartile, whiskers represent lower and higher bound ±1.5× interquartile range. All Wilcoxon tests shown here (paired or unpaired) were two sided.

**Extended Data Fig. 4:**
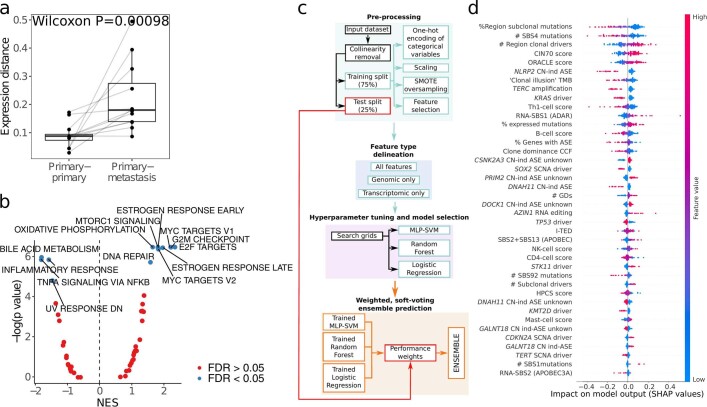
Transcriptional features of metastasis. **a**. Expression distance between paired primary tumour regions; compared to distance between paired primary and non-LN intrathoracic metastatic tumour regions. Only patients with two or more primary regions and at least one metastatic region sampled are shown (12 primary-metastasis pairs from 8 tumours). Boxes represent lower quartile, median and upper quartile, whiskers represent lower/higher bound +/− 1.5 x interquartile range. Significance was tested using a paired Wilcoxon test (P = 0.00098). **b**. Gene set enrichment analysis (GSEA) of functional groups from hallmark gene sets^14^ between metastasis seeding and non-seeding regions. Only tumours where both seeding and non-seeding regions had RNA-seq were included (n = 37, 122 regions). Dots coloured by a significant enrichment after FDR correction. Mean normalised enrichment score (NES) is displayed on the x-axis and indicates the enrichment for a given gene set, and the negative log of the adjusted P value is displayed on the y-axis. **c**. Overview schematic of the machine learning framework used to predict whether a region contains a metastasis-seeding clone(s). MLP-SVM: multilayer-perceptron with support vector machine terminal layer. **d**. Individual Shapley Additive Explanations (SHAP) values for the most important features across the combined ensemble. Positive SHAP values indicate weighting towards a prediction of metastasis seeding whereas negative SHAP values indicate a weighting towards prediction of metastasis non-seeding. Colour scale represents the value of the feature across the test dataset (red=high values, blue=low values). For instance, high values of the ORACLE expression marker (red dots) were associated with a higher likelihood of a region being seeding (positive SHAP values) in the combined ensemble. The predictions were based on 516 primary tumour regions from 206 tumours where seeding status could be established and where all metrics tested could be measured (307 non-seeding regions, 209 seeding), with a 75%-25% training-test dataset split. TMB: tumour mutational burden; CN-ind ASE: Copy number-independent allele specific expression; HPCS: High Plasticity Cell State^5^; GD: genome doubling; CCF: cancer cell fraction; Clone dominance CCF: maximum CCF at terminal nodes of a phylogenetic tree; SCNA: somatic copy number alteration.

Finally, we evaluated the dynamics of transcriptomic diversity during metastatic progression. We observed significantly higher transcriptomic diversity between paired primary–metastatic tumour regions than between primary regions derived from the same tumour (Fig. 4a). This relationship remained consistent when considering only intrathoracic non-LN metastases (Extended Data Fig. 4a), which suggests that there are consistent differences between primary and metastatic transcriptomes that cannot be fully explained by microenvironmental differences between metastatic organ sites. To further explore this finding, we compared transcriptomic diversity between metastasis-seeding or non-seeding primary tumour regions and their paired metastatic tumours (Fig. 4b). Across the cohort, expression patterns in metastases were more similar to the metastasis-seeding primary regions than non-seeding primary regions (*n* = 22 primary–metastasis pairs from 18 tumours^6^; *P* = 0.0019, two-tailed paired Wilcoxon test; Fig. 4c). Gene set enrichment analyses between these regions showed an enrichment within seeding regions for gene sets linked to proliferation and a depletion in immune-linked groups^14^ (Extended Data Fig. 4b). Taken together, these results suggest that a proportion of the transcriptomic patterns observed in metastatic tumours are underpinned by somatic changes that originated in the primary tumour and are capable of influencing ongoing evolution, including metastasis.

To further explore this result, we evaluated the impact of relevant molecular features on the metastatic potential of a tumour region. In particular, we tested whether transcriptomic features are informative for inferring metastatic potential. To achieve this, we built an ensemble machine-learning classifier to predict whether a tumour region contained a seeding clone (or seeding clones) (Methods and Extended Data Fig. 4c). Leveraging a recently published approach^33^, we defined three feature sets: genomic only; transcriptomic only; and combined genomic and transcriptomic (see Methods for details on initial feature selection). For each feature set, we trained three model types (logistic regression, random forest, multilayer perceptron with support vector machine terminal layer) and selected the best model in each case (hyperparameter tuning using a randomized search grid across relevant parameters with *K*-fold stratified cross validation; the best model was selected as that with the highest balanced accuracy — see Methods for full details). The combined genomic and transcriptomic feature set generated a marginally better classifier relative to the classifiers generated from the independent genomic and transcriptomic feature sets (Fig. 4d, combined receiver operator characteristic (ROC) area under the curve (AUC) = 0.79; genomic only ROC AUC = 0.69, transcriptomic only ROC AUC = 0.73). Overall, the combined feature classifier showed promising performance with an accuracy of 71% (significantly greater than the no-information rate, *P* = 0.0007), although with much greater specificity than sensitivity (sensitivity = 0.51, specificity = 0.86). Of the variables tested, the two most important to infer metastatic potential related to the evolutionary context of the mutations within the tumour region: a decreased proportion of subclonal mutations that were present in only a subset of tumour cells within the tumour region (that is, were not regionally dominant)^7^ and a decreased number of mutations linked to the smoking signature SBS4 (likely a proxy for the trunk length of the phylogenetic tree)^34,35^ (Fig. 4e and Extended Data Fig. 4d). Similarly, increased CIN70 (ref. ^36^) and ORACLE^17^ expression signature scores, both associated with proliferation, were also associated with metastatic potential and demonstrated strong weighting across different models. Other variables described in this work also helped the classifier to discriminate regions with metastatic potential. These included CN-independent ASE within *NLRP2*, RNA-SBS1 activity and the proportion of genes with CN-independent ASE per region.

## Discussion

Multiregion sequencing studies in the past decade have highlighted important genomic alterations in cancer, including point mutations and CN alterations, that drive ITH and fuel cancer evolution^2,3,37–39^.

Through paired genomic–transcriptomic analysis and multiregion sampling of NSCLC, we highlight sources of variation that would be missed by an exclusively genomic approach. Highly expressed cancer genes with low intratumour variance were more likely to be under positive selection. This finding could inform studies seeking to discover new cancer genes. Our results also imply the presence of limited yet significant negative selection in cancer evolution, consistent with constraints to tumour development^16,40^. The additional resolution gained by restricting to uniformly expressed genes mirrors results reported in a previous publication, in which an expression signature composed of such genes represented a robust biomarker in NSCLC^17^.

We find pervasive ASE in NSCLC and find that, as expected in a disease characterized by significant chromosomal instability, in the majority of tumours, ASE is predominantly explained by SCNAs. However, we highlight an important fraction of ASE that is linked to epigenomic dysfunction, in particular to changes in DNA methylation and inactivating mutations in the histone methyltransferase *SETD2*, the lysine demethylase *KDM5C* and the lysine methyltransferase *KMT2B* genes. Our observations build on in vivo single-cell studies of mouse models of LUAD, which have highlighted the importance of a dynamic epigenome in governing tumour progression^41^. Furthermore, our results provide orthogonal insight into the role of these genes in transcription: loss of *SETD2* has been linked to increased oncogenic transcriptional output^42^, and *KDM5C* regulates transcription through H3K4 demethylation^43^. In addition, previous work has linked ASE to epigenomic changes through enhancer activity^44^. Future work should focus on the interplay between mutated epigenetic modifiers and enhancer activity in cancer.

We also utilize approaches that have previously been leveraged to define DNA mutational signatures to extract unbiased trinucleotide signatures of RNA single-base substitutions from paired DNA and RNA-seq. We show that these signatures underlie RNA editing. Importantly, two signatures, RNA-SBS1 (linked to ADAR editing) and RNA-SBS2 (linked to APOBEC3A editing) seemed to be underpinned by heritable somatic mechanisms. That is, their activities were preserved between paired primary and metastatic samples, which is consistent with recent work linking genomic variants with RNA editing^45^. Their potential importance to tumour evolution is underlined by the roles that APOBEC enzymes play in driving genome instability^46^ and by the observed relationship between RNA-SBS1 activity and patterns of aberrant hypomethylation (Extended Data Fig. 3f). *ADAR*, which we suggest underpins RNA-SBS1, has been proposed as a tumour suppressor gene that is essential to cancer cells in the context of epigenomic dysfunction owing to its ability to target otherwise immunogenic double-stranded RNAs within short interspersed nuclear elements^32^. Furthermore, our data strongly suggests that *APOBEC3A* drives the observed RNA-SBS2 signature, consistent with a stronger role for *APOBEC3A* compared with other *APOBEC3* gene family members, including *APOBEC3B* in RNA^47,48^.

Multiregion paired genomic–transcriptomics enables characterization of the phenotype of the metastasis-seeding region of the primary tumour. To elucidate the influence of transcriptomic features on the biology of metastasis, we used a combined machine-learning approach, which revealed key features of the metastatic transition and demonstrated that both genomic and transcriptomic features are able to predict metastatic potential. In particular, we found that two genomic metrics relating to the evolutionary context of mutations (proxies for the dominance of a subclone within a tumour region and decreased phylogenetic trunk length) and two gene expression signatures related to proliferation were markers of metastatic potential. In a companion paper^7^, we report that the presence of recent subclonal expansions (that is, large subclones at the terminus of a phylogenetic branch) are associated with shorter disease-free survival. We also observed that within both circulating tumour DNA and primary tumour tissue, the size of subclonal mutational clusters is linked to their metastatic potential^6,49^. Conceivably, recent subclonal expansions might be driven by increased proliferation, which is captured by transcriptomic signatures. Also, for a given tumour region, metrics related to the evolutionary background of its constituent mutations were associated with metastatic potential. The finding that such metrics tended to be more useful at differentiating primary tumour regions with and without metastasis-seeding potential than the specific genes in which mutations occurred may have important implications for biomarker discovery.

Of note, this machine-learning approach combined multiple variables, which together might render some features redundant within the classifier. This could mean that variables not presented within Fig. 4e might be biologically important.

A limitation of this work is that it is unlikely to have captured the true extent of transcriptomic variation in these tumours. We did not consider all forms of transcriptional variation, including alternative splicing. Furthermore, we could only study ASE in the minority of genes that contain an expressed SNP, and we applied a strict, but specific, approach to defining CN-independent ASE. Similarly, the filters applied to identify RNA variants, rather than transcribed DNA mutations, were stringent, and only exonic events (capturing only a fraction of RNA editing)^26^ were considered. Therefore, it is likely that we have underestimated the variation and biological impact attributable to these processes in tumours. Nevertheless, in this way, we were able to identify previously unknown RNA-SBS signatures, including three of unknown aetiology. This result highlights the need for further studies that examine patterns of RNA editing in larger datasets. Finally, our machine-learning approach utilized tumour region transcriptomic data that was not deconvolved at the subclone level. Therefore we could not test subclone-specific metrics, which are also likely to affect metastatic potential^6^.

Despite these limitations, this work has shown that transcriptional variation is likely to play an important part in NSCLC evolution. It has revealed sources of diversity that would not have been identified by a focused analysis of the cancer genome and underlined the importance of multi-omic sequencing and systems biology approaches to the study of tumour evolution.

## Methods

### Data generation and processing

#### TRACERx cohort

The TRACERx study (ClinicalTrials.gov identifier NCT01888601) is a prospective observational cohort study that aims to transform our understanding of NSCLC, the design of which has been approved by an independent research ethics committee (13/LO/1546). Informed consent for entry into the TRACERx study was mandatory and obtained from every participant. All participants were assigned a study identity number that was known to the individual. These were subsequently converted to linked study identities such that the participants could not identify themselves in study publications. All human samples (tissue and blood) were linked to the study identity number and barcoded such that they were anonymized and tracked on a centralized database, which was overseen by the study sponsor only.

The cohort in this manuscript includes the fraction of samples from the first 421 participants (described in detail in two companion manuscripts^6,7^) with RNA-seq data available after quality checking before and after sequencing (CONSORT diagram in the Supplementary Information).

Seven samples from individuals with disease relapse (CRUK0046_BR_T1-R1, CRUK0046_BR_T1-R2, CRUK0069_MR_T1-R1, CRUK0069_MR_T1-R2, CRUK0280_BR_T1-R1, CRUK0280_BR_T1-R2 and CRUK0679_BP_T1-R1) were not associated with any primary tumour, and one normal sample (CRUK0643_SU_N01) was not paired with any tumour sample with RNA-seq data. These eight samples are present in the raw data but were not included in downstream analyses. Additionally, for four LN samples (CRUK0099_SU_LN01, CRUK0227_SU_LN01, CRUK0240_SU_LN01 and CRUK0240_SU_LN02) the seeding tumour region could not be established^6^, and these samples were therefore not used in paired primary–metastatic analyses.

#### RNA extraction and sequencing

For each sample, total RNA was extracted using a dual extraction method for RNA and DNA using AllPrep DNA/RNA Mini kits (Qiagen). Frozen samples were transferred onto cold Petri dishes kept on dry ice and dissected into 20–30 mg samples. Before extraction, the freshly dissected tissue was transferred directly to homogenization tubes with RLT plus lysis buffer. Homogenization of tissues was carried out using a TissueRuptor II probe or bead methods and by passing the lysate through a QIAshredder column (Qiagen). Libraries were prepared using a minimum of 100 ng input of total RNA, where possible, using an Illumina TruSeq Stranded Total RNA Human/Mouse/Rat ribo-depletion library preparation kit (20020597), and PCR was amplified for 15 cycles according to the manufacturer’s guidelines. Final libraries were quality checked using Agilent Tapestation and Promega QuantiFluor dsDNA and were then pooled in equimolar solutions. Sequencing was performed using an Illumina HiSeq 4000 platform at a depth of 50 million paired reads per sample, with a length of 75 bp or 100 bp per read.

#### DNA methylation sequencing

A subset of previously published primary NSCLC data of the first 100 participants of the TRACERx study with multiple tumour regions were selected for RRBS^21^.

The NuGEN Ovation RRBS Methyl-Seq system adapted for automation was applied by using 100 ng of gDNA per sample, digested with MspI, ligated to sequencing adapters and processed using Qiagen’s EpiTect Fast DNA Bisulfite kit. Bisulfite-converted DNA libraries were then amplified by PCR (12 cycles) and purified using Agencourt RNAClean XP magnetic beads. The quantity and quality of the resulting libraries were evaluated using a Qubit dsDNA HS assay (Invitrogen) and an Agilent Bioanalyzer High Sensitivity DNA assay (Agilent Technologies), respectively. Samples were multiplexed in pools of 8 and sequenced on a HiSeq2500 system in single-end 100 bp runs. Sequencing outputs were converted into fastq files. FastQC (v.0.11.2)^51^ was used for quality control, and Trim Galore! (Babraham Institute), a wrapper around Cutadapt^52^, was applied with default settings to perform quality and adapter trimming for each set of paired-end fastq files. The bisulfite-converted DNA sequence aligner Bismark (v.0.14.4)^53^ was used to align reads to the UCSC reference genome hg19 build, and PCR deduplication was carried out using NuDup, leveraging NuGEN’s molecular tagging technology (v.2.3; https://github.com/nugentechnologies/nudup).

In total, 96 tumour and 31 tumour-adjacent normal regions from 31 patients (average 3 tumour regions per patient) had matched RRBS and RNA-seq data available.

#### RNA-seq alignment and gene expression

Illumina adapters were trimmed from raw sequencing reads using Cutadapt (v.2.10)^52^ with standard parameters, and the quality of the trimmed reads were estimated per flow cell lane using FastQC (v.0.11.9)^51^. Only fastq files with less than 80% of total reads being duplicates were kept for alignment. Fastq read files passing these quality checks were aligned to the UCSC hg19 human reference genome build using STAR (v.2.5.2a)^54^ in two-pass mode with ENCODE 3 parameters, generating one BAM file per tumour region. The same reads were also mapped to the human transcriptome (RefSeq GCA_000001405.1 build) using the same STAR parameters to generate gene expression data. Next, we marked duplicates using the MarkDuplicates function from GATK (v.4.1.7.0)^55^. Aligned reads were quality checked using QoRTs (v.1.3.6)^56^ to assess RNA integrity. Somalier (v.0.2.7)^57^ was used to detect potential instances of sample mislabelling and FastQ Screen (0.14.0)^58^ was used to detect potential instances of contamination. FastQC, QoRTs and Somalier outputs were visualized using MultiQC (v.1.9)^59^. RSEM (v.1.3.3)^60^ was used with default parameters to quantify gene expression from the BAM files aligned to the transcriptome. Gene expression patterns were used for further quality checking of each sample. Tumour regions with >40% of all genes with zero counts (estimated using the QoRTS output Genes_WithZeroCounts) were excluded. Additionally, samples with <20% of reads mapping to a genomic area covered by exactly one gene in a coding sequence genomic region (estimated using the QoRTS output ReadPairs_UniqueGene_CDS) were excluded. Next, RNA coverage was calculated for single nucleotide variants (SNVs) detected in matched whole-exome sequencing data per tumour region using SAMtools (v.1.9)^61^ mpileup. Mutation expression was used to further quality check the mapping of RNA reads. The expression of SNVs exclusive to a given tumour region was used to detect potential instances of within-patient mislabelling of RNA–DNA matched tumour regions as well as to exclude normal adjacent lung tissue regions that expressed mutations present in paired tumour regions. A similar approach was applied to germline SNPs to further assess potential sample swaps based on patterns of CN variation from matched DNA per tumour region. Tumour regions in which fewer than 10 mutations, or fewer than 25% of the total mutation count, had evidence of expression, and/or less than 10% of SNPs had evidence of biallelic expression, were excluded. Finally, tumour regions clustering with tumour-adjacent normal tissue regions (see the section ‘UMAP clustering’) and tumour regions with a low purity were also excluded from further analyses. To ensure the reproducibility and portability of the above pipeline, all steps described were implemented through the Nextflow (v.20.07.1)^62^ pipeline manager.

### Analyses

Unless explicitly specified otherwise, all Wilcoxon tests performed in this work are two-sided, using the function wilcox.test() in base R. To account for the effect of each individual tumour when comparing tumour regions in the cohort, we use linear mixed-effects models throughout the manuscript. These were fitted using the package lmerTest (v.3.1-3)^63^ in R, using the parent tumour from which the tumour region was derived as a random effect. Significance was obtained comparing a null model with the model containing the variable of interest using the base R function anova(), and setting refit = TRUE to test the impact of fixed effects.

Unless stated otherwise, plots were generated in the R environment (v.3.6.3), using ggplot2 (v.3.2.1)^64^, ggpubr (v.0.4.0), cowplot (v.1.0.0), scales(v.1.0.0) and ggrepel (v.0.8.1).

#### Gene expression distance

RSEM raw read counts were first normalized using the median of ratios method implemented in DESeq2 (v.1.24.0)^65^. Genes with more than 5 read counts in at least 20% of the cohort (a total of 20,136 genes) were kept after filtering. Variant stabilizing transformation (VST) was performed on the normalized reads. Distance correlation was calculated for each sample to all other samples in the cohort for the top 500 most variable genes using the dcor() function from the R package energy (v.11.7-6)^66^. In total, 500 was chosen as the number of variable genes to keep, as previous tests showed that this number represented the variance in the cohort while reducing the computational resources needed to calculate a cohort wide correlation distance. The correlation distance provides a measure from 0 (no similarity) to 1 (maximum similarity); to transform this metric into distance, we subtracted the resulting distance correlation from 1. Primary tumour regions were then clustered on the basis of the minimum distance to other samples across the cohort.

#### UMAP clustering

VST counts from all samples in the cohort were used to generate a UMAP^67^ of expression patterns across the cohort. UMAP was performed using the umap (v.2.7.7.0) package in R with default parameters.

#### LUAD drivers within non-LUAD NSCLCs

Samples that were considered to fall within the LUAD cluster (UMAP 1<2.5 and UMAP 2>0) were evaluated for an enrichment in driver mutations more commonly associated with LUADs.

Differential mutation analysis was conducted to establish driver alterations that were enriched in LUADs relative to non-LUAD NSCLCs. For each driver mutation (a total of 266 genes were considered; see the section ‘Epigenetic drivers’ and a companion manuscript^7^ for how these mutations were annotated), a Fisher’s exact test was performed comparing the numbers of non-LUAD and LUAD tumour regions that harboured the mutation with those that did not. After adjusting for repeated measures using the Benjamini–Hochberg method^68^, the four genes in which driver mutations were significantly enriched among LUADs compared with non-LUADs were *KRAS*, *EGFR*, *STK11* and *RBM10*. A Fisher’s exact test was then performed to test the relative enrichment of these ‘LUAD-favoured’ events within non-LUADs clustering with LUADs in the UMAP compared with non-LUADs not clustering with LUADs in the UMAP.

Although these non-LUAD tumours had been subjected to independent histological review, we further reviewed histological features of these tumours in terms of morphological heterogeneity and immunohistochemistry staining profiles, including TTF-1, p63 and p40, CD56, synaptophysin and chromogranin, and pan-cytokeratin to confirm the histological diagnosis and to investigate the presence and extent of the adenocarcinomatous components in these non-LUAD tumours.

#### PCA

A PCA was performed using VST counts for tumour regions extracted at surgery, in LUAD and LUSC tumours separately. PCA was performed using the prcomp() function in base R, centring the data but not scaling, as expression data had already been scaled through VST. The PCs adding up to 30% of variance explained in LUAD and LUSC separately were subjected to further analysis.

Each PC was then linked at the tumour region level with multiple genomic and clinical features using a linear-mixed effects model accounting for the tumour from which the regions were derived and tumour purity as a covariate. PC ITH was calculated as the standard deviation for each PC divided by the median PC value. The selection of features is described in the section ‘PCA feature selection’.

#### RNA ASCAT

Estimates of tumour fraction were calculated using the tumour purity values from ASCAT^50^. The RNA-derived estimates of tumour fraction (referred to in the main text as tumour transcript fraction) were calculated using a modified version of ASCAT. In brief, using this approach, at SNP sites, the B allele frequency (BAF) was calculated using the non-duplicated RNA-seq reads aligning to each allele using SAMtools (v.1.9)^61^ mpileup. This RNA BAF was then used as input to ASCAT instead of DNA BAF, whereas DNA-derived logR was maintained. The RNA-derived estimate of tumour fraction was the estimated tumour purity when ploidy was identical to that of the DNA.

#### I-TED

To estimate ITH, we focussed only on tumours with more than one region sampled (813 samples from 280 tumours). Because standard measures of heterogeneity might be affected by the number of regions available per tumour, a pairwise approach was used to estimate ITH. For each region of a tumour, 1 minus the correlation distance between VST gene expression to all other regions in the same tumour was calculated for the top 500 most variable genes. The correlation distance was calculated using the function dcor() in the R package energy (v.1.7-6)^66^. Only genes with read counts above 5 in at least 20% of the cohort (a total of 20,136 genes) were considered for this analysis. Tumour-level I-TED was calculated as the median I-TED of all regions. This metric was independent of the number of regions sampled (Fig. 1c).

The relationship between I-TED and purity, CN and mutation heterogeneity as well as histological subtype and number of regions per tumour was tested using a multivariable linear regression. The percentage of variance explained by each type of alteration was calculated using the Anova function from the R package car (v.3.0-6)^69^.

Mutational and CN heterogeneity were calculated based on metrics from a companion manuscript^7^. In brief, for CN heterogeneity, the total proportion of region-specific CN events compared with the total number of CN events was determined. For mutation heterogeneity, the proportion of subclonal mutations at the tumour level was obtained compared with the total number of mutations per tumour. Both metrics were bootstrapped by resampling to account for differences in the number of regions samples per tumour.

#### Differential gene expression and gene set enrichment analyses

All differential gene expression and subsequent gene set enrichment analyses (GSEAs) were performed using the following approach. First, trimmed mean of *M*-values normalization from the edgeR (v.3.26.5)^70^ R package was performed on RSEM raw counts. Genes with expression below 30 counts per million in <70% of the smallest group size were removed using the function filterByExpr() with min.count set to 30. Expression differences were performed at the region level through the limma-voom analytical pipeline, taking tumour as a blocking factor, by performing within-tumour expression correlations and including them within the voom model estimate using the duplicateCorrelation() function. This method is analogous to using tumour as a random effect in a linear mixed-effects model. The raw *P* values provided by limma for differential expression were then corrected for multiple testing using the Benjamini–Hochberg (FDR) method^68^.

The *t*-statistic generated by limma was used as input for GSEA for MSigDB hallmark gene sets^14^ using the R package fgsea (v.1.10.1)^71^ with default parameters.

#### ASE analysis

To understand patterns of ASE in the TRACERx cohort, we focused on genes containing an expressed heterozygous SNP and quantified the number of unique reads aligning to each parental allele with a minimum mapping quality of 0 and a minimum base quality score of 13 using SAMtools (v.1.9)^61^ mpileup. Only SNPs with a total coverage of greater than eight such reads were considered to be expressed. Further filtering removed SNPs in blacklisted regions of the genome with poor mappability. The blacklisted genomic regions were obtained from UCSC Genome Table Browser and include regions excluded from the ENCODE project (both DAC and Duke list), simple repeats, segmental duplications and microsatellite regions^72^.

In accordance with the expected distribution of allelic expression, a beta-binomial test was used to test for ASE^73^ using the pbetabinom function from the R package VGAM (v.1.1-2)^74^ and an overdispersion parameter *σ* of 0.05. We attribute allele-specific RNA reads to the major and minor alleles, as inferred from multiregion DNA-derived allele-specific CN data. Specifically, for each SNP, the allele with the greatest number of reads in the corresponding whole-exome DNA sequencing (DNA-seq) data was considered the major allele. RNA-seq reads reporting this allele were designated major allele RNA reads and vice versa for the minor allele RNA reads.

To assess the probability of obtaining allele-specific read counts at least as disparate as the observed distribution, given an expected allelic expression ratio of 0.5, the following beta-binomial (Betabin) test was performed with the following parameters:

1 $${\rm{Betabin}}(X\ge m,t,0.5)$$

where *m* represents the major allele RNA reads and *t* the total RNA reads at that heterozygous SNP. To alleviate the multiple testing burden and preserve statistical power, we performed independent filtering by using the following binomial test criterion and retaining only those heterozygous SNPs for which *P* < 0.001:

2 $${\rm{Bin}}(X\ge t,t,{\rm{CPNratio}}){\rm{;}}P < 0.001$$

where *t* is the total RNA reads at that heterozygous SNP and CPNratio is the raw major allele copy number divided by the total copy number at that site. In effect, this removes sites with low read counts and/or extreme CN ratios such as regions with loss of heterozygosity or high-level allele-specific amplifications.

To test whether ASE was CN-dependent or CN-independent, two further beta-binomial tests were performed using the following parameters:

3 $${\rm{Betabin}}(X < m,t,0.5)$$

4 $${\rm{Betabin}}(X\ge m,t,{\rm{CPNratio}})$$

where *m* again represents the major allele RNA-seq read count, *t* the total RNA reads at that heterozygous SNP, and CPNratio the raw local major allele CN divided by the total CN. Following this, two combined *P* values were generated from all SNPs within each gene using the Fisher method: one (A) using the *P* value from test equation (1); and the second (B) using the smallest *P* value from either test equation (3) or equation (4). The Benjamini–Hochberg approach was used to adjust for multiple hypothesis testing across all genes considered. Genes with an adjusted *P* value (FDR) < 0.05 from test equation (1) but not either equation (3) or (4) were considered to show CN-dependent ASE, whereas those with an adjusted *P* value < 0.05 from either test equation (3) or (4) were considered to show CN-independent ASE. The adjusted *P* value threshold of <0.05 for either one of two one-tailed tests was chosen given the stringency of this approach to investigate CN-independent ASE.

The RNA allelic ratio can vary between 0.5 and the CPNratio, given the tumour allele-specific CN, owing to expression levels in the tumour and admixed non-tumour cells. This approach therefore in effect tests for ASE beyond that which would be seen given expression only from the tumour, or non-tumour component, of the bulk sample, accounting for the estimated CN status of the gene of interest within the tumour.

Combining point mutation and insertion–deletion calls within each gene and their corresponding gene ASE classifications, we computed the Fisher exact test statistics of the odds of observing each mutation type listed at ASE versus non-ASE genes, within genes in which we were powered to detect ASE (that is, containing an expressed heterozygous SNP).

We also considered the potential impact of reference bias, whereby heterozygous sites are incorrectly assigned as being homozygous as a result of the use of a generic reference genome, potentially resulting in false-positive ASE calls, on our results. To account for this, we quantified the extent to which reference bias was present in each sample. For each sample, the total instances of CN-independent ASE in which the reference allele was overexpressed relative to the alternative allele was divided by the total instances in which the reverse was true. To ensure this phenomenon was not affecting our results, we tested the impact of adding this per-sample quantification of reference bias as an additional covariate to the linear mixed-effects models within Fig. 2d,e. The statistical associations presented in those figures remained consistent after this additional test.

#### ASM analysis

To evaluate the relationship between ASM and expression, we leveraged previously published CAMDAC pure tumour methylation rates, *m*_t_, derived from multiregion bulk tumour and adjacent normal RRBS performed on a subset of TRACERx samples^21^. We subset these methylomes to promoter-associated CpGs in CpG islands with read depths of ≥10 in the adjacent-matched normal sample and at the promoters of genes with CN-dependent or CN-independent ASE information in at least one sample. In total, 11,254 genes met these criteria. On average, 4,345 and 2,771 genes had promoter methylation information and could be tested for CN-dependent and CN-independent ASE per sample, respectively.

For each sample, genes were classified with respect to their CN-dependent and CN-independent ASE test statistics. Genes with *P* < 0.05 were deemed as ASE and those with *P* > 0.5 as not significantly ASE. In the case of CN-dependent ASE, genes were required to show no significant ASE, irrespective of CN, to be categorized as not significantly ASE. Genes with no phasing information were not tested for ASE.

Previously reported findings^21^ indicated that intermediate *m*_t_ signals are in large part due to subclonal ASM. We therefore used intermediate CAMDAC *m*_t_ values as a proxy for ASM. CpGs with methylation rates 99% highest density intervals (HDI^99^) ⊆ [0.15,0.75] and point estimates ${\epsilon }$ [0.2, 0.7] were deemed ASM. We required three consecutive ASM loci to classify a gene promoter as ASM.

Combining promoter ASM and corresponding gene ASE classifications, we computed Fisher’s exact test statistics of the odds of observing ASM at ASE versus non-ASE genes, within genes in which we were powered to detect ASE (that is, containing an expressed heterozygous SNP).

#### Tumour–normal differential methylation analysis

For a subset of TRACERx samples with previously published RRBS data, we obtained a list of tumour–normal differentially methylated positions based on CAMDAC *m*_t_ values and using the tumour-adjacent normal methylation rate as a proxy for the cell of origin, *m*_n_ (*P* < 0.01 and |*m*_t_ *–* *m*_n_| > 0.2). For each of these, we computed the number of CpGs that were significantly hypomethylated and hypermethylated in tumour samples compared to the normal samples, taking only loci that had coverage in all samples (min_normal_ = 10, min_tumour_ = 3). We then calculated the fraction of differentially methylated positions that were hypomethylated. Using a linear mixed effects model, with tumour identity as random effect, we then compared this metric to the percentage of genes showing evidence of CN-independent ASE per sample (separately for LUAD and LUSC).

#### ASE PCA and imputation

PCA was performed to test for differences in patterns of CN-independent ASE between tumour and normal tissue samples. Only genes in which it was possible to test for CN-independent ASE (that is, having an expressed SNP that was not in a region of extreme CN) in at least 100 samples across the cohort were considered. The negative natural logarithm of the FDR from the test for CN-independent ASE in the section ‘ASE analysis’ for each gene was computed. In samples for which it was not possible to test for CN-independent ASE for a given gene, the median negative natural logarithm for that gene in all tumour and normal tissue samples across the cohort was imputed. Data were scaled and centred, and PCA performed using the function prcomp() in base R.

#### Epigenetic drivers

A list of epigenetic modifier genes was obtained from previous work^22^. We collated a cancer gene list out of all genes identified in the COSMIC cancer gene census (v.75)^75^, supplemented with those identified in large-scale pan-cancer analyses (using FDR < 0.05 as cut-off)^76^ and previous large-scale NSCLC sequencing studies^77–79^ (this list is also utilized in a companion paper^7^). Genes overlapping the epigenetic modifier and cancer gene lists (see the companion paper^7^ for the definition of cancer genes in our cohort) were considered. Any non-silent variant located within one of these genes underwent further categorization on the basis of the following criteria: if the mutation was found to be deleterious (either a stop-gain or predicted deleterious in two of the three computational approaches applied (Sift^80^, Polyphen^81^ and MutationTaster^82^)) and the gene was annotated as being recessive by COSMIC (tumour suppressor), the variant was classified as a driver mutation. Also, if the gene was annotated as being dominant (oncogene) by COSMIC and we could identify ≥3 exact matches of the specific variant in COSMIC, it was classified as a putative driver mutation. Frequently mutated genes, containing more than five putative driver mutations across the cohort, were incorporated into the below model.

A univariable linear-mixed effects model using region-level mutation and CN-independent ASE data was run to establish the effect of driver mutations in individual genes on the proportion of genes tested showing CN-independent ASE. *P* values were adjusted for repeated measures using the Benjamini–Hochberg method^68^. Independent predictors were defined using a multivariable linear-mixed effects model, using all epigenetic modifiers taking the tumour containing the region as a random factor.

#### Validation of link between *SETD2*-inactivating mutation and CN-independent ASE with cell line data

A literature search was conducted to find publicly available cell line data with which it would be possible to test the impact of *SETD2*, *KDM5C* or *KMT2B* knockdown or knockout after ASE in an isogenic human setting.

Three separate relevant publications^23–25^ were identified for *SETD2*, only one for *KDM5C*^83^ and none for *KMT2B*. Therefore, we proceeded to focus solely on the impact of *SETD2* on CN-independent ASE. This was done in lung cells (H1650; three biological replicates with shRNA knockdown^23^), kidney cells (786-0; single replicate with ZFN knockout^24^) and liver cells (HepG2; single replicate with CRISPR-mediated knockout^25^).

In each study, DNA-seq was not performed alongside RNA-seq, and it was therefore not possible to obtain a highly accurate and contemporaneous record of heterozygous sites across the genome (used to measure ASE) and CN events. Conceivably, both of these sources of variation might fluctuate with passaging. A proxy was therefore sought: SNPs and SCNAs listed within the Cancer Cell Line Encyclopaedia^84^ (in the case of H1650 and 786-0); or within another publication (in the case of HepG2, SNPs were derived from variant calling of whole-genome sequencing data^85^). To mitigate the possibility of false-positive ASE calls arising from inaccurate genotyping and subsequent misclassifying of homozygous sites as heterozygous, a site was only considered as heterozygous if it was both annotated as such in the relevant DNA-seq study while also harbouring at least one expressed read from both alleles within the RNA-seq data. At sites of DNA allelic imbalance, the allele with the majority of available RNA reads assigned to it was considered likely to be the major allele. With this record of heterozygous sites, analysis of CN-independent ASE was performed in the same way as described in section ‘ASE analysis’. Finally, the impact of *SETD2* on CN-independent ASE was evaluated using a linear mixed-effects model, with the study added as a random factor to control for the additional biological replicates within ref. ^23^.

#### Detection of RNA variants

RNA-specific variants were called using the somatic variant caller Mutect2 from GATK (v.4.1.7.0)^55,86^. Each BAM file was first pre-processed following GATK’s best practices for RNA-variant calling. In brief, marked duplicated reads were removed and splice junctions split followed by a base quality recalibration to ensure the compatibility of the mapped reads with GATK’s variant callers. The somatic variant caller Mutect2 was then run to generate raw putative RNA variant calls in exonic regions only, integrating information from multiple regions per tumour, using the multiple-sample mode. To filter germline variants, a blood DNA sample was added as a ‘normal’ region per tumour, along with GATK’s panel of normal samples based on DNA-seq from 4,136 normal samples from The Cancer Genome Atlas and Genome Aggregation Database (gnomAD) sites. The option-tumour-lod-to-emit was set to 2.0 to ensure a maximum number of raw calls. Variant calls were run per chromosome in parallel using GNU parallel (v.20210422)^87^. FilterMutectCalls was then used to filter the raw calls with the additional option -read-filter NotSupplementaryAlignmentReadFilter to exclude variants supported exclusively by supplementary reads. After this first filtering step, BCFtools (v1.10.2)^88^ was run to select only PASS biallelic SNVs.

Next, bam-readcount (v.0.8)^89^ was used to obtain RNA reads with a base and mapping quality above 20 supporting the variants called by Mutect2 as an orthogonal measure of variant calls at these sites. On the basis of the bam-readcount output, the following criteria were applied to remove variants: variants with fewer than 30 reads in the germline DNA; with fewer than 30 reads in total for all DNA tumour regions; with an RNA coverage below 10 reads; for which the alternative base was supported by fewer than 3 reads; or present at less than 1% variant allele frequency. Additionally, further filtering was applied to variants in regions of the genome with poor mappability such as centromeres, repetitive regions, genomic regions with high nucleotide variability in the sample. These included blacklisted genomic regions obtained from UCSC Genome Table Browser, excluded from the Encode project (both DAC and Duke list)^72^ as well as regions coding for immunoglobulin antibodies in hg19: chromosome 14, positions beyond 106000000, chromosome 22 between positions 22385572 and 23265082, and chromosome 2 between positions 132032200 and 133174000. In positions at which the RNA variant was supported by one or more reads from the DNA tumour samples, support in the DNA might arise from sequencing errors in very high coverage regions. To distinguish between this scenario and expressed mutations, a one-tailed Fisher’s test was performed comparing the number of DNA reads supporting the RNA variant to the number of reads supporting other variants compared to the total coverage  at the same position. If the number of DNA reads supporting the RNA variant was distinguishable from sequencing noise (with a non-stringent *P* value threshold of 0.1), the RNA variant was excluded. Furthermore, variants flanked by the same four nucleotides as either the reference or alternative allele were also excluded.

Because the libraries used for RNA-seq were stranded, variant reads from each strand were compared to obtain the difference in strandedness relative to the total depth at the variant position.

Additionally, we selected ten putative editing events for Sanger sequencing, all of which were validated with this orthogonal method (Extended Data Table 2).

#### RNA-SBS signatures

The R package hdp (v.0.1.5)^90^, available on GitHub (https://github.com/nicolaroberts/hdp), was used to call de novo RNA-SBS signatures using default parameters and 15 iterations using the trinucleotide context of strand-independent variants (192 possibilities in total). To prevent sample size biases for which RNA variants present in all regions in highly sampled tumours could be artificially over-represented during de novo signature calling, we ran this step using unique RNA variants  across all samples per patient. Only de novo signatures with significant exposure (as determined by hdp) in at least 1% of the cohort were considered for further analyses. A signature present in only one patient was therefore discarded for downstream analyses. deconstructSigs (v1.9.0)^91^ was then applied to each individual sample to estimate RNA-SBS signatures per sample. Only tumour regions with more than 20 RNA variants were considered for further analyses.

To test the potential relationship between signature activity and the expression of specific genes, we performed a linear mixed-effects model using the number of RNA variants attributed to each signature as the dependent variable, and gene expression of all genes in our dataset (*n* = 20,136). Expression was measured as log_10_(transcripts per million + 1) for genes with at least 5 read counts in 20% of the RNA-seq cohort.

To further test the relationship between *APOBEC* expression and RNA-SBS2, a linear mixed-effects model was performed, using the number of RNA variants attributed to RNA-SBS2 as the dependent variable, the expression of all *APOBEC* genes in the transcriptome as explanatory variables and the tumour identifier as a random effect.

#### Detection of RNA loops

RNA loops were detected in the flanking regions of RNA variants. Flanking regions were derived using the flanks() function from the R package GenomicRanges (v.1.36.0)^92^. Loops were defined by the flanking regions 3′ and 5′ of a 3–5-nucleotide-long sequence containing an RNA variant being complementary at a length of at least 3 nucleotides.

#### RNA-editing motif enrichment

To confirm the role of specific *APOBEC* enzymes in RNA-SBS2, we tested the relative proportions of C>T events at known RNA-editing *APOBEC* motifs. *APOBEC* enzymes typically edit C>T variants at the fourth position of 4-nucleotide-long RNA hairpin loops. In particular, *APOBEC3A* favours the CAT[C>T] motif^30,31^.

*APOBEC* motif enrichment analyses were performed based on a previously reported local enrichment method^93^. In brief, for each C>T variant site, a Fisher’s test was performed to test whether C>T changes within 20 upstream or downstream nucleotides occurred more than expected by chance at specific motifs (CAT[C>T]) in either strand.

#### ITH of CN-independent ASE

The ITH of CN-independent ASE was calculated for each tumour as follows. The total number of genes in a tumour showing CN-independent ASE in all of two or more tumour regions was divided by the total number of genes in that tumour showing CN-independent ASE in at least two regions. A gene showed homogeneous CN-independent ASE if it was detected in all regions of a tumour for which it was possible to test as outlined in the section ‘ASE analysis’.

The relationship between I-TED and the ITH of other forms of alterations was tested using a multivariable linear regression in a similar fashion as that detailed in the section ‘I-TED’.

#### dN/dS analysis

The dndscv function in R from the dNdScv package (v.0.1.0.0)^16^ was run on all mutations available in the cohort. The function genesetdnds() was then run on the resulting object on various subsets of gene lists divided by expression quantiles for ITH, intertumour heterogeneity or amplitude. This ensured that the global dN/dS metrics obtained for each group were based on the same mutational background, making them more comparable.

Expression amplitude was measured as VST counts, whereas ITH was measured as the standard deviation in expression amplitude across all regions in multiregion tumours. Intertumour heterogeneity was measured as the bootstrapped (ten iterations) standard deviation in expression per gene sampling one tumour region per tumour per iteration, as in the previously described^17^.

Cancer genes were defined as specified in^7^ the section ‘Epigenetic drivers’ as well as in a companion manuscript^7^. Non-cancer genes were those not present in the pan-cancer COSMIC database (v.75)^75^ or the list of cancer genes from ref. ^94^. Essential genes were identified from the Project Achilles list of essential genes for NSCLC^18^.

#### PCA feature selection

The following clinical and genomic features per primary tumour region were tested for association with the foremost PC of gene expression:

1. Clinical features, including age of the patient, sex, years spent smoking cigarettes and TNM stage of the primary tumour at resection. See methods in a companion manuscript^7^ for details on how these features were obtained.
2. LUAD-specific subtype as defined by central pathological review (acinar, lepidic, cribriform, micropapillary, mucinous, papillary or solid). This feature was available only for LUAD tumours and is described in more detail in a companion paper^95^.
3. Tumour mutation burden: the number of mutations per region. Only mutations that are likely to have a phenotypic effect are included, in line with calculations of a harmonized tumour mutation burden^96^. These include all exonic single-nucleotide mutations, except synonymous changes, as well as insertions and deletions. All metrics below that depend on mutation numbers are based on this set of mutations.
4. Presence or absence of driver mutations in cancer genes with a driver mutation in at least 5% of the cohort. This included driver mutations in *ARID1A*, *ATM*, *ATRX*, *CDKN2A*, *COL5A2*, *CREBBP*, *EGFR*, *FAT1*, *KEAP1*, *KMT2D*, *KRAS*, *MGA*, *NF1*, *PIK3CA*, *RBM10*, *SMARCA4*, *STK11* and *TP53*. See a companion manuscript^7^ on the definition of cancer genes in our cohort.
5. Proportion of subclonal mutations: the number of exonic mutations in the focal tumour region belonging to subclonal mutational clusters in the tumour, divided by the total number of exonic mutations in that region. Subclonal mutations were defined as those belonging to any mutation cluster with a cancer cell fraction below 1 across the tumour (that is, not present in all cells in the focal tumour). Details on how clonal clusters are determined are available from a companion manuscript^7^. This metric gives a measure of the proportion of smaller clones present in the tumour region.
6. Genome instability at the tumour region level, a common feature in tumour evolution^38^, was measured through the weighted genome instability index (wGII), which measures the extent of genome instability per tumour region. See methods in a companion manuscript^7^ for details on the calculation of this index.
7. Similarly, the number of whole genome duplication events per tumour region was also considered. See methods in a companion manuscript^7^ for details on the calculation of genome doubling events.
8. COSMIC mutational signatures SBS1, SBS2, SBS4, SBS5, SBS13 and SBS92 (ref. ^34^). Signature activity was measured as the fraction of mutations per tumour region corresponding to each signature’s weight. SBS2 and SBS13 were combined into a single SBS DNA signature for APOBEC activity. See methods in a companion manuscript^7^ for details on the mutational signature analysis.
9. The immune microenvironment was assessed by estimating the T cell fraction from DNA using the R package T-Cell ExTRECT(v.1.0.1)^97^.

Additionally, we performed a single sample GSEA (ssGSEA) for the 50 MSigDB hallmark gene sets using the R package fgsea (v.1.10.1)^71^ on VST counts using a Gaussian distribution and default parameters. The resulting enrichment scores per sample were correlated to each PC using a linear mixed-effects model that controlled for the tumour of origin. The resulting *P* values were merged by MSigDB functional group^14^ (Extended Data Table 3) using the harmonic mean and corrected for multiple testing using FDR.

#### Classifier feature selection

The seeding region classifier was based on a cohort of regions from primary tumours that had metastasized or that had not metastasized after 3 years of follow up. Only tumour regions with a seeding clone at >0.2 CCF were considered as seeding for this analysis. In total, 516 primary tumour regions from 206 tumours for which seeding status could be established and for which all metrics tested could be measured (307 non-seeding regions, 209 seeding) were analysed. The following features were also considered for the classifier:

1. Tumour mutation burden: the number of mutations per region. Only mutations that are likely to have a phenotypic effect are included, in line with calculations of a harmonized tumour mutation burden^96^. These include all exonic single-nucleotide mutations, except synonymous changes, as well as insertions and deletions. All metrics below that depend on mutation numbers are based on this set of mutations.
2. Regionally truncal and clonal mutation burden: the number of clonal mutations per tumour region. Clonal mutations were defined as those belonging to a mutation cluster with a cancer cell fraction of 1 (that is, present in all cells) in the tumour region. This included mutations that are clonal in the entire tumour (trunk mutations) as well as mutations that were clonal in the focal region, but subclonal or absent in other regions of the same tumour. See methods in a companion manuscript^7^ for details on the determination of the truncal cluster.
3. Clonal illusion tumour mutation burden: the number of mutations that were clonal in the focal region, but not clonal within all other regions of the same tumour. Only mutations belonging to clusters with a cancer cell fraction of 1 (that is, present in all tumour cells) in the focal region, but not in the rest of the tumour, were counted. See methods in a companion manuscript^7^ for more details on the definition of the truncal cluster.
4. Proportion of regionally subclonal mutations: the number of mutations belonging to subclonal mutational clusters in the focal tumour region divided by the total number of mutations in that region. Subclonal mutations were defined as those belonging to any mutation cluster with a cancer cell fraction below 1 (that is, not present in all cells in the focal tumour region). Details on how clonal clusters were determined are available in a companion paper^7^. This metric gives a measure of the proportion of smaller clones present in the tumour region.
5. Proportion of expressed mutations: the number of expressed mutations divided by the total mutation burden in the tumour region. A mutation is considered expressed if it had at least three reads with the mutated allele in the RNA-seq data. This metric serves as a proxy for the proportion of tumour mutations that were present in the bulk RNA-seq transcripts.
6. Number of region clonal driver mutations: the number of driver mutations that belong to clonal mutation clusters in the focal region. This included both truncal mutations (that is, clonal across the tumour) and clonal illusion mutations (that is, clonal in the focal region but not in the rest of tumour regions). Details on how driver mutations and clonal clusters were determined are available in a companion manuscript^7^.
7. Number of region subclonal driver mutations: the number of driver mutations that belong to subclonal mutation clusters (cancer cell fraction below 1) in the focal region. Details on how driver mutations and clonal clusters were determined are available in a companion manuscript^7^.
8. Presence or absence of driver mutations in cancer genes that contained a driver mutation in at least 10% of the cohort. This included driver mutations in *CDKN2A*, *KEAP1*, *KMT2D*, *KRAS*, *SMARCA4*, *STK11* and *TP53*.
9. Presence or absence of CN drivers, that is, amplification of a subset of oncogenes or the homozygous loss of a subset of tumour suppressor genes. Genes with at least copy number driver alterations in 10% of the cohort were included. These include *SOX2, TERT, TERC, CDKN2A, MYC, CCND1, FGFR1, NKX2-1, AKT2, EGFR* and *CCNE1*. Details on how driver mutations and clonal clusters are determined are available in a companion paper^7^.
10. COSMIC mutational signatures SBS1, SBS2, SBS4, SBS5, SBS13 and SBS92 (ref. ^34^). Signature activity was measured as the fraction of mutations per tumour region corresponding to each signature’s weight. SBS2 and SBS13 were combined into a single SBS DNA signature for APOBEC activity. Details on how mutational signatures were extracted are available in a companion manuscript^7^.
11. Genome instability at the tumour region level, a common feature in tumour evolution^38^, was measured through the wGII, which measures the extent of genome instability per tumour region. Details on how this metric was calculated per tumour region are available in a companion paper^7^.
12. Similarly, the number of genome-doubling events per tumour region was also considered. Details on how genome-doubling events per tumour region were calculated are available in a companion paper^7^.
13. In a companion paper^7^, the presence of expanded subclones in a tumour were taken as evidence of recent subclonal sweeps and linked to poor prognosis. To test the potential impact of this metric on seeding potential, per tumour region, we calculated the maximum cancer cell fraction of all mutation clusters on terminal nodes of the phylogenetic tree  as a measure of clone dominance. A higher cancer cell fraction indicates a larger terminal mutation cluster in the focal region. Details on how driver mutations and clonal clusters are determined are available in the companion paper^7^.
14. To measure the impact of expression diversity within a tumour, we included the per tumour region I-TED score. I-TED was imputed as the median score across the cohort for samples for which only one region per tumour was available.
15. To characterize the phenotype of the tumour region, we measured the tumour-region enrichment score through ssGSEA (using the R package fgsea (v.1.10.1)^71^ using a Gaussian distribution and default parameters on VST counts) for three cancer-specific gene sets: (1) CIN70 (ref. ^36^): an expression signature linked to genome instability and cell proliferation, phenotypes that have been linked to poor prognosis and metastasis^98^; (2) Oracle^17^: a lung cancer-specific prognostic maker, in which increased expression of this gene set is linked to poor prognosis; (3) a high-plasticity cell state: an expression signature for phenotypic plasticity extracted from the recent publication from ref. ^5^. biomaRt (v.2.40.1)^99^ 1:1 orthologues between human and mice genes from cluster 5 were used to calculate this signature, as described in the publication.
16. The tumour microenvironment was characterized using expression markers consistent with previously described immune cell types^100^.
17. Additionally, tumour purity as calculated using ASCAT (v.2.3)^50^ was included.
18. To test the potential effect of overall tumour-specific expression in the metastatic potential, we added the differential between the transcript tumour fraction (described in section ‘RNA ASCAT’) and tumour purity as calculated using ASCAT from DNA.
19. We tested the potential impact of CN-independent ASE on the metastatic potential of tumour regions by including the proportion of genes with CN-independent ASE compared with the total number of genes for which ASE could be measured per tumour region.
20. We also included the ASE status of genes with significant enrichment in CN-independent ASE in tumours compared to tumour-adjacent normal lung tissue. These included *CSN2KA3*, *DNAH11*, *DOCK1*, *GALNT18*, *NLRP2*, *PRIM2* and *ZNF597*. In cases when ASE could not be measured in a tumour region, the ASE status was encoded as unknown to prevent missing values.
21. The potential impact of RNA editing on seeding potential was included through the two RNA-editing signatures characterized in this paper: RNA-SBS1 (ADAR) and RNA-SBS2 (APOBEC3A). Their activity was measured as the fraction of RNA variants per tumour region corresponding to each signature’s weight.
22. We additionally included the RNA-editing levels (fraction of RNA molecules with edited sites) of three genes reported to play a role in cancer development from the literature^101–103^: *AZIN1*, *COPA* and *COG3*. This feature was added only for tumour regions with at least 30 unique RNA reads covering the editing sites of interest.

#### Classifier to predict seeding and non-seeding tumour regions

We built the machine-learning framework in Python using Tensorflow (v.2.6.0)^104^ and sklearn (v.0.0)^105^. Specifically, we built an ensemble classifier that used three different model types: (1) logistic regression, (2) random forest and (3) multilayer perceptron with support vector machine embedded in the final layer. We describe the structure of the machine-learning pipeline in more detail below.

##### Pre-processing

To pre-process the input data, we first explored the correlation structure among potential explanatory features (*n* = 61) and removed those features with high correlation coefficients (*r* > 0.75, *n* = 11). We one-hot-encoded categorical features using get_dummies from Pandas (v1.3.3)^106^ and then split the data into training and test datasets (75/25 split). After encoding, we had a total of 60 features. We scaled the continuous features using MinMaxScaler from sklearn.preprocessing (v.0.0)^107^ and used SMOTENC from imblearn.over_sampling (v.0.8.0)^105^ to improve the balance of the dataset in terms of numbers of seeding and non-seeding regions. Finally, we used the sklearn (v.0.0)^105^ framework to perform additional variable selection before training using a LinearSVC model (penalty = “l1”), keeping those features with importance ≥0.015. This threshold removed 15 out of 60 features. Following this initial pre-processing, we generated different subsets of the dataset depending on the source of the input features, thus downstream processes within the pipeline operated on three datasets: (1) genomic only features, (2) transcriptomic only features, and (3) all features.

##### Model training

For each model type, to tune model hyperparameters, we performed a randomized grid search with RandomizedSearchCV (sklearn.model_selection, v.0.0)^104^ and StratifiedKFold cross-validation (n_splits = 10, n_iter = 500).

We implemented a sequential model from tensorflow.keras (v.2.6.0)^104^ with dropout layers (dropout = 0.2) to reduce overfitting and used a categorical hinge loss function with an l2 kernel regularizer and sigmoid activation function in the final layer. This approach effectively constitutes a support vector machine in the final layer of the sequential model. We used the Adam optimizer from tensorflow.keras.optimizers (v.2.6.0)^104^. Specifically, we defined a search grid to tune the following parameters: learning rate, batch size, epochs, number of hidden layers and sizes of hidden layers. Following the cross-validated training across the randomized search grid, we selected the best performing model according to the greatest balanced accuracy. We then extracted feature weights from this selected model using PermutationImportance from eli5.sklearn (v.0.11.0). Finally, to assess the performance of the selected model on the held-out test dataset, we used the model to predict whether a test region was seeding or not and compared this to the true labels. The machine-learning pipeline was developed using Python (v.3.5.5), and plots of results were generated in R (v.4.0.3) using ggplot2 (v.3.3.5).

### Reporting summary

Further information on research design is available in the Nature Portfolio Reporting Summary linked to this article.

## Online content

Any methods, additional references, Nature Portfolio reporting summaries, source data, extended data, supplementary information, acknowledgements, peer review information; details of author contributions and competing interests; and statements of data and code availability are available at 10.1038/s41586-023-05706-4.

## Supplementary information

Supplementary Information Supplementary file containing the CONSORT diagram detailing which samples from the TRACERx 421 whole-exome sequencing cohort were used for RNA-seq.

Reporting Summary

## Figures and Tables

**Extended Data Table 1:**
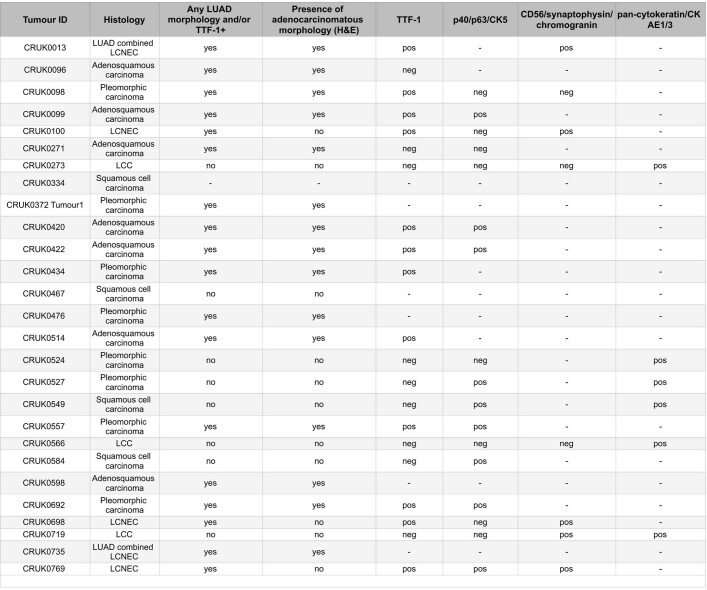
Central pathological review of non-LUAD tumours clustering with LUADs based on expression patterns

Histological subtype was determined by centralized pathological slide review, as was the presence of adenocarcinomatous morphology within these tumours. Additionally, immunohistochemical staining profiles were summarized according to the pathology reports. LCC, large cell carcinoma; LCNEC, large cell neuroendocrine carcinoma.

**Extended Data Table 2:**
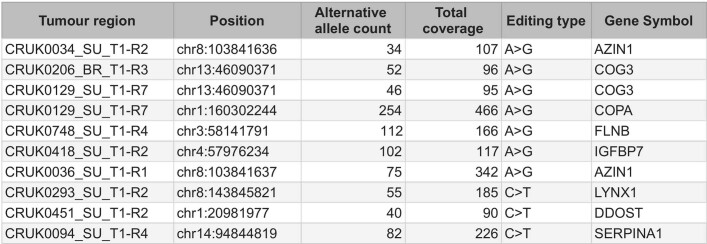
Sanger sequencing-validated RNA variant sites

RNA variant sites detected through our bioinformatics pipeline, and validated using Sanger sequencing. The variant was detected in the RNA but not the DNA of the same tumour region.

**Extended Data Table 3:**
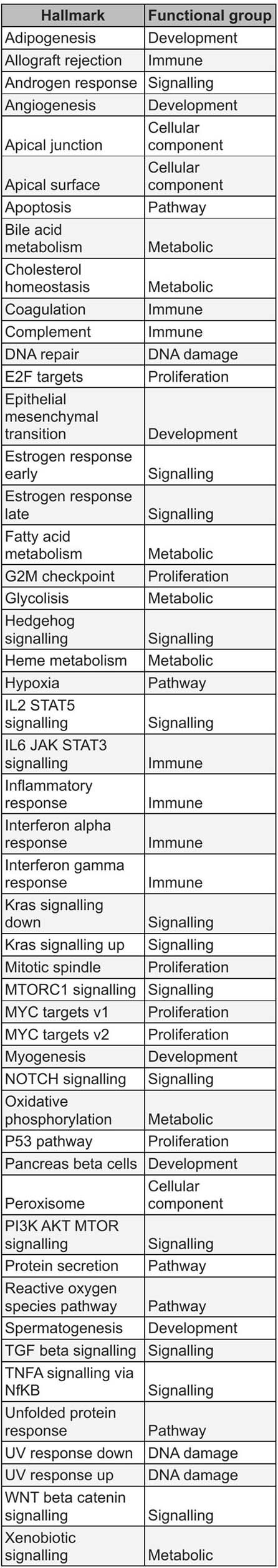
Hallmark gene set functional groups

MSigDB hallmark gene set and their functional group as assigned by the authors^14^.

## Data Availability

The RNA-seq, whole-exome sequencing and RRBS data (in each case from the TRACERx study) used during this study have been deposited at the European Genome–phenome Archive, which is hosted by the European Bioinformatics Institute and the Centre for Genomic Regulation, under the accession codes EGAS00001006517 (RNA-seq), EGAS00001006494 (whole-exome sequencing) and EGAS00001006523 (RRBS). Access is controlled by the TRACERx data access committee. Details on how to apply for access are available at the linked page.

## Code availability

Code used to process data and generate figures is available at the following link: 10.5281/zenodo.7603386.

## References

[1] Black, J. R. M. & McGranahan, N. Genetic and non-genetic clonal diversity in cancer evolution. *Nat. Rev. Cancer* **21**, 379–392 (2021). 33727690 10.1038/s41568-021-00336-2

[2] Bailey, C. et al. Tracking cancer evolution through the disease course. *Cancer Discov.* **11**, 916–932 (2021). 33811124 10.1158/2159-8290.CD-20-1559 PMC7611362

[3] Jamal-Hanjani, M. et al. Tracking the evolution of non-small-cell lung cancer. *N. Engl. J. Med.* **376**, 2109–2121 (2017). 28445112 10.1056/NEJMoa1616288

[4] PCAWG Transcriptome Core Group et al. Genomic basis for RNA alterations in cancer. *Nature* **578**, 129–136 (2020). 32025019 10.1038/s41586-020-1970-0 PMC7054216

[5] Marjanovic, N. D. et al. Emergence of a high-plasticity cell state during lung cancer evolution. *Cancer Cell* **38**, 229–246.e13 (2020). 32707077 10.1016/j.ccell.2020.06.012 PMC7745838

[6] Al Bakir, M. et al. The evolution of non-small cell lung cancer metastases in TRACERx. *Nature*10.1038/s41586-023-05729-x (2023). 10.1038/s41586-023-05729-x PMC10115651 37046095

[7] Frankell, A. M. et al. The evolution of lung cancer and impact of subclonal selection in TRACERx. *Nature*10.1038/s41586-023-05783-5 (2023). 10.1038/s41586-023-05783-5 PMC10115649 37046096

[8] Rosenthal, R. et al. Neoantigen-directed immune escape in lung cancer evolution. *Nature* **567**, 479–485 (2019). 30894752 10.1038/s41586-019-1032-7 PMC6954100

[9] Travis, W. D. et al. The 2015 World Health Organization classification of lung tumors: impact of genetic, clinical and radiologic advances since the 2004 classification. *J. Thorac. Oncol.* **10**, 1243–1260 (2015). 26291008 10.1097/JTO.0000000000000630

[10] Lam, V. K. et al. Targeted tissue and cell-free tumor DNA sequencing of advanced lung squamous-cell carcinoma reveals clinically significant prevalence of actionable alterations. *Clin. Lung Cancer* **20**, 30–36.e3 (2019). 30279110 10.1016/j.cllc.2018.08.020

[11] East, P. et al. Oncogenic RAS activity predicts response to chemotherapy and outcome in lung adenocarcinoma. *Nat. Commun.* **13**, 5632 (2022). 10.1038/s41467-022-33290-0 PMC9512813 36163168

[12] Dogan, S. et al. Molecular epidemiology of *EGFR* and *KRAS* mutations in 3,026 lung adenocarcinomas: higher susceptibility of women to smoking-related KRAS-mutant cancers. *Clin. Cancer Res.* **18**, 6169–6177 (2012). 23014527 10.1158/1078-0432.CCR-11-3265 PMC3500422

[13] Buettner, R. Invasive mucinous adenocarcinoma: genetic insights into a lung cancer entity with distinct clinical behavior and genomic features. *Mod. Pathol.* **35**, 138–139 (2022). 34795416 10.1038/s41379-021-00945-0 PMC8786656

[14] Liberzon, A. et al. The Molecular Signatures Database (MSigDB) hallmark gene set collection. *Cell Syst.* **1**, 417–425 (2015). 26771021 10.1016/j.cels.2015.12.004 PMC4707969

[15] Cao, S. et al. Estimation of tumor cell total mRNA expression in 15 cancer types predicts disease progression. *Nat. Biotechnol.* **40**, 1624–1633 (2022). 10.1038/s41587-022-01342-x PMC9646498 35697807

[16] Martincorena, I. et al. Universal patterns of selection in cancer and somatic tissues. *Cell* **171**, 1029–1041.e21 (2017). 29056346 10.1016/j.cell.2017.09.042 PMC5720395

[17] Biswas, D. et al. A clonal expression biomarker associates with lung cancer mortality. *Nat. Med.* **25**, 1540–1548 (2019). 31591602 10.1038/s41591-019-0595-z PMC6984959

[18] Cowley, G.S. et al. Parallel genome-scale loss of function screens in 216 cancer cell lines for the identification of context-specific genetic dependencies. *Sci. Data* **1**, 140035 (2014). 25984343 10.1038/sdata.2014.35 PMC4432652

[19] Castel, S. E., Levy-Moonshine, A., Mohammadi, P., Banks, E. & Lappalainen, T. Tools and best practices for data processing in allelic expression analysis. *Genome Biol.* **16**, 195 (2015). 26381377 10.1186/s13059-015-0762-6 PMC4574606

[20] Baran, Y. et al. The landscape of genomic imprinting across diverse adult human tissues. *Genome Res.* **25**, 927–936 (2015). 25953952 10.1101/gr.192278.115 PMC4484390

[21] Larose Cadieux, E. et al. Copy number-aware deconvolution of tumor-normal DNA methylation profiles. Preprint at *bioRxiv*10.1101/2020.11.03.366252 (2020).

[22] Feinberg, A. P., Koldobskiy, M. A. & Göndör, A. Epigenetic modulators, modifiers and mediators in cancer aetiology and progression. *Nat. Rev. Genet.* **17**, 284–299 (2016). 26972587 10.1038/nrg.2016.13 PMC4888057

[23] Zhou, Y. et al. Histone methyltransferase SETD2 inhibits tumor growth via suppressing CXCL1-mediated activation of cell cycle in lung adenocarcinoma. *Aging* **12**, 25189–25206 (2020). 33223508 10.18632/aging.104120 PMC7803529

[24] Ho, T. H. et al. High-resolution profiling of histone H3 lysine 36 trimethylation in metastatic renal cell carcinoma. *Oncogene* **35**, 1565–1574 (2016). 26073078 10.1038/onc.2015.221 PMC4679725

[25] Chen, K. et al. Methyltransferase SETD2-mediated methylation of STAT1 is critical for interferon antiviral activity. *Cell* **170**, 492–506.e14 (2017). 28753426 10.1016/j.cell.2017.06.042

[26] Bazak, L. et al. A-to-I RNA editing occurs at over a hundred million genomic sites, located in a majority of human genes. *Genome Res.* **24**, 365–376 (2014). 24347612 10.1101/gr.164749.113 PMC3941102

[27] Picardi, E., D’Erchia, A. M., Lo Giudice, C. & Pesole, G. REDIportal: a comprehensive database of A-to-I RNA editing events in humans. *Nucleic Acids Res.* **45**, D750–D757 (2017). 27587585 10.1093/nar/gkw767 PMC5210607

[28] Sharma, S., Patnaik, S. K., Taggart, R. T. & Baysal, B. E. The double-domain cytidine deaminase APOBEC3G is a cellular site-specific RNA editing enzyme. *Sci. Rep.* **6**, 39100 (2016). 27974822 10.1038/srep39100 PMC5156925

[29] Teh, Y. W., Jordan, M. I., Beal, M. J. & Blei, D. M. Hierarchical dirichlet processes. *J. Am. Stat. Assoc.* **101**, 1566–1581 (2006).

[30] Sharma, S. & Baysal, B. E. Stem-loop structure preference for site-specific RNA editing by APOBEC3A and APOBEC3G. *PeerJ* **5**, e4136 (2017). 29230368 10.7717/peerj.4136 PMC5723131

[31] Sharma, S. et al. APOBEC3A cytidine deaminase induces RNA editing in monocytes and macrophages. *Nat. Commun.* **6**, 6881 (2015). 25898173 10.1038/ncomms7881 PMC4411297

[32] Mehdipour, P. et al. Epigenetic therapy induces transcription of inverted SINEs and ADAR1 dependency. *Nature* **588**, 169–173 (2020). 33087935 10.1038/s41586-020-2844-1

[33] Sammut, S.-J. et al. Multi-omic machine learning predictor of breast cancer therapy response. *Nature* **601**, 623–629 (2022). 34875674 10.1038/s41586-021-04278-5 PMC8791834

[34] Alexandrov, L. B. et al. The repertoire of mutational signatures in human cancer. *Nature* **578**, 94–101 (2020). 32025018 10.1038/s41586-020-1943-3 PMC7054213

[35] Alexandrov, L. B. et al. Signatures of mutational processes in human cancer. *Nature* **500**, 415–421 (2013). 23945592 10.1038/nature12477 PMC3776390

[36] Carter, S. L., Eklund, A. C., Kohane, I. S., Harris, L. N. & Szallasi, Z. A signature of chromosomal instability inferred from gene expression profiles predicts clinical outcome in multiple human cancers. *Nat. Genet.* **38**, 1043–1048 (2006). 16921376 10.1038/ng1861

[37] Yates, L. R. et al. Subclonal diversification of primary breast cancer revealed by multiregion sequencing. *Nat. Med.* **21**, 751–759 (2015). 26099045 10.1038/nm.3886 PMC4500826

[38] Watkins, T. B. K. et al. Pervasive chromosomal instability and karyotype order in tumour evolution. *Nature* **587**, 126–132 (2020). 32879494 10.1038/s41586-020-2698-6 PMC7611706

[39] Landau, D. A. et al. Mutations driving CLL and their evolution in progression and relapse. *Nature* **526**, 525–530 (2015). 26466571 10.1038/nature15395 PMC4815041

[40] López, S. et al. Interplay between whole-genome doubling and the accumulation of deleterious alterations in cancer evolution. *Nat. Genet.* **52**, 283–293 (2020). 32139907 10.1038/s41588-020-0584-7 PMC7116784

[41] LaFave, L. M. et al. Epigenomic state transitions characterize tumor progression in mouse lung adenocarcinoma. *Cancer Cell* **38**, 212–228.e13 (2020). 32707078 10.1016/j.ccell.2020.06.006 PMC7641015

[42] Xie, Y. et al. SETD2 loss perturbs the kidney cancer epigenetic landscape to promote metastasis and engenders actionable dependencies on histone chaperone complexes. *Nat. Cancer* **3**, 188–202 (2022). 35115713 10.1038/s43018-021-00316-3 PMC8885846

[43] Outchkourov, N. S. et al. Balancing of histone H3K4 methylation states by the Kdm5c/SMCX histone demethylase modulates promoter and enhancer function. *Cell Rep.* **3**, 1071–1079 (2013). 10.1016/j.celrep.2013.02.030 23545502

[44] Sungalee, S. et al. Histone acetylation dynamics modulates chromatin conformation and allele-specific interactions at oncogenic loci. *Nat. Genet.* **53**, 650–662 (2021). 33972799 10.1038/s41588-021-00842-x

[45] Li, Q. et al. RNA editing underlies genetic risk of common inflammatory diseases. *Nature* **608**, 569–577 (2022). 35922514 10.1038/s41586-022-05052-x PMC9790998

[46] Venkatesan, S. et al. Induction of APOBEC3 exacerbates DNA replication stress and chromosomal instability in early breast and lung cancer evolution. *Cancer Discov.* **11**, 2456–2473 (2021). 33947663 10.1158/2159-8290.CD-20-0725 PMC8487921

[47] Jalili, P. et al. Quantification of ongoing APOBEC3A activity in tumor cells by monitoring RNA editing at hotspots. *Nat. Commun.* **11**, 2971 (2020). 32532990 10.1038/s41467-020-16802-8 PMC7293259

[48] Petljak, M. et al. Mechanisms of *APOBEC3* mutagenesis in human cancer cells. *Nature* **607**, 799–807 (2022). 35859169 10.1038/s41586-022-04972-y PMC9329121

[49] Abbosh, C. et al. Tracking early lung cancer metastatic dissemination in TRACERx using ctDNA. *Nature*10.1038/s41586-023-05776-4 (2023). 10.1038/s41586-023-05776-4 PMC7614605 37055640

[50] Van Loo, P. et al. Allele-specific copy number analysis of tumors. *Proc. Natl Acad. Sci. USA* **107**, 16910–16915 (2010). 20837533 10.1073/pnas.1009843107 PMC2947907

[51] Andrews, S. et al. FastQC v0.11.2 (Babraham Bioinformatics, 2010).

[52] Martin, M. Cutadapt removes adapter sequences from high-throughput sequencing reads. *EMBnet.J* **17**, 10–12 (2011).

[53] Krueger, F. & Andrews, S. R. Bismark: a flexible aligner and methylation caller for bisulfite-seq applications. *Bioinformatics* **27**, 1571–1572 (2011). 21493656 10.1093/bioinformatics/btr167 PMC3102221

[54] Dobin, A. et al. STAR: ultrafast universal RNA-seq aligner. *Bioinformatics* **29**, 15–21 (2013). 23104886 10.1093/bioinformatics/bts635 PMC3530905

[55] McKenna, A. et al. The Genome Analysis toolkit: a MapReduce framework for analyzing next-generation DNA sequencing data. *Genome Res.* **20**, 1297–1303 (2010). 20644199 10.1101/gr.107524.110 PMC2928508

[56] Hartley, S. W. & Mullikin, J. C. QoRTs: a comprehensive toolset for quality control and data processing of RNA-seq experiments. *BMC Bioinformatics* **16**, 224 (2015). 26187896 10.1186/s12859-015-0670-5 PMC4506620

[57] Pedersen, B. S. et al. Somalier: rapid relatedness estimation for cancer and germline studies using efficient genome sketches. *Genome Med.* **12**, 62 (2020). 32664994 10.1186/s13073-020-00761-2 PMC7362544

[58] Wingett, S. W. & Andrews, S. FastQ Screen: A tool for multi-genome mapping and quality control. *F1000Res.* **7**, 1338 (2018). 10.12688/f1000research.15931.1 PMC6124377 30254741

[59] Ewels, P., Magnusson, M., Lundin, S. & Käller, M. MultiQC: summarize analysis results for multiple tools and samples in a single report. *Bioinformatics* **32**, 3047–3048 (2016). 27312411 10.1093/bioinformatics/btw354 PMC5039924

[60] Li, B. & Dewey, C. N. RSEM: accurate transcript quantification from RNA-seq data with or without a reference genome. *BMC Bioinformatics* **12**, 323 (2011). 21816040 10.1186/1471-2105-12-323 PMC3163565

[61] Li, H. et al. The Sequence Alignment/Map format and SAMtools. *Bioinformatics* **25**, 2078–2079 (2009). 19505943 10.1093/bioinformatics/btp352 PMC2723002

[62] Di Tommaso, P. et al. Nextflow enables reproducible computational workflows. *Nat. Biotechnol.* **35**, 316–319 (2017). 28398311 10.1038/nbt.3820

[63] Kuznetsova, A., Brockhoff, P. B. & Christensen, R. H. B. lmerTest package: tests in linear mixed effects models. *J. Stat. Softw.*10.18637/jss.v082.i13 (2017).

[64] Wickham, H. *ggplot2: Elegant Graphics for Data Analysis*. (Springer International Publishing, 2016).

[65] Love, M. I., Huber, W. & Anders, S. Moderated estimation of fold change and dispersion for RNA-seq data with DESeq2. *Genome Biol.* **15**, 550 (2014). 25516281 10.1186/s13059-014-0550-8 PMC4302049

[66] Rizzo, M. L. & Szekely, G. J. energy: E-Statistics: multivariate inference via the energy of data. R package version 1.7-0 (2017).

[67] McInnes, L., Healy, J. & Melville, J. UMAP: uniform manifold approximation and projection for dimension reduction. Preprint at *arXiv*10.48550/arXiv.1802.03426 (2018).

[68] Benjamini, Y. & Hochberg, Y. Controlling the false discovery rate: a practical and powerful approach to multiple testing. *J. R. Stat. Soc.* **57**, 289–300 (1995).

[69] Fox, J. & Weisberg, S. *An R Companion to Applied Regression* (SAGE Publications, 2018).

[70] Robinson, M. D., McCarthy, D. J. & Smyth, G. K. edgeR: a Bioconductor package for differential expression analysis of digital gene expression data. *Bioinformatics* **26**, 139–140 (2010). 19910308 10.1093/bioinformatics/btp616 PMC2796818

[71] Sergushichev, A. A. An algorithm for fast preranked gene set enrichment analysis using cumulative statistic calculation. Preprint at *bioRxiv*10.1101/060012 (2016).

[72] Amemiya, H. M., Kundaje, A. & Boyle, A. P. The ENCODE blacklist: identification of problematic regions of the genome. *Sci. Rep.* **9**, 9354 (2019). 31249361 10.1038/s41598-019-45839-z PMC6597582

[73] Skelly, D. A., Johansson, M., Madeoy, J., Wakefield, J. & Akey, J. M. A powerful and flexible statistical framework for testing hypotheses of allele-specific gene expression from RNA-seq data. *Genome Res.* **21**, 1728–1737 (2011). 21873452 10.1101/gr.119784.110 PMC3202289

[74] Yee, T. W., Stoklosa, J. & Huggins, R. M. The VGAM package for capture-recapture data using the conditional likelihood. *J. Stat. Softw.* **65**, 1–33 (2015).

[75] Forbes, S. A. et al. COSMIC: exploring the world’s knowledge of somatic mutations in human cancer. *Nucleic Acids. Res.* **43**, D805–D811 (2015). 25355519 10.1093/nar/gku1075 PMC4383913

[76] Lawrence, M. S. et al. Discovery and saturation analysis of cancer genes across 21 tumour types. *Nature* **505**, 495–501 (2014). 24390350 10.1038/nature12912 PMC4048962

[77] Cancer Genome Atlas Research Network. Comprehensive genomic characterization of squamous cell lung cancers. *Nature* **489**, 519–525 (2012). 22960745 10.1038/nature11404 PMC3466113

[78] Collisson, E. et al. Comprehensive molecular profiling of lung adenocarcinoma: the Cancer Genome Atlas Research Network. *Nature* **511**, 543–550 (2014). 25079552 10.1038/nature13385 PMC4231481

[79] Campbell, J. D. et al. Distinct patterns of somatic genome alterations in lung adenocarcinomas and squamous cell carcinomas. *Nat. Genet.* **48**, 607–616 (2016). 27158780 10.1038/ng.3564 PMC4884143

[80] Kumar, P., Henikoff, S. & Ng, P. C. Predicting the effects of coding non-synonymous variants on protein function using the SIFT algorithm. *Nat. Protoc.* **4**, 1073–1081 (2009). 19561590 10.1038/nprot.2009.86

[81] Adzhubei, I., Jordan, D. M. & Sunyaev, S. R. Predicting functional effect of human missense mutations using PolyPhen-2. *Curr. Protoc. Hum. Genet.* **7**, Unit 7.20 (2013). 10.1002/0471142905.hg0720s76 PMC4480630 23315928

[82] Schwarz, J. M., Rödelsperger, C., Schuelke, M. & Seelow, D. MutationTaster evaluates disease-causing potential of sequence alterations. *Nat. Methods* **7**, 575–576 (2010). 20676075 10.1038/nmeth0810-575

[83] Shen, H. F. et al. The dual function of KDM5C in both gene transcriptional activation and repression promotes breast cancer cell growth and tumorigenesis. *Adv. Sci.* **8**, 2004635 (2021). 10.1002/advs.202004635 PMC8097366 33977073

[84] Ghandi, M. et al. Next-generation characterization of the Cancer Cell Line Encyclopedia. *Nature* **569**, 503–508 (2019). 31068700 10.1038/s41586-019-1186-3 PMC6697103

[85] Zhou, B. et al. Haplotype-resolved and integrated genome analysis of the cancer cell line HepG2. *Nucleic Acids Res.* **47**, 3846–3861 (2019). 30864654 10.1093/nar/gkz169 PMC6486628

[86] Benjamin, D. et al. Calling somatic SNVs and indels with Mutect2. Preprint at *bioRxiv*10.1101/861054 (2019).

[87] Tange, O. GNU Parallel 2018. *Zenodo*10.5281/zenodo.1146014 (2018).

[88] Danecek, P. et al. Twelve years of SAMtools and BCFtools. *Gigascience* **10**, giab008 (2021). 33590861 10.1093/gigascience/giab008 PMC7931819

[89] Khanna, A. et al. Bam-readcount–rapid generation of basepair-resolution sequence metrics. Preprint at https://arxiv.org/abs/2107.12817 (2021). 10.21105/joss.03722 PMC13367885 42453900

[90] Li, Y. et al. Patterns of somatic structural variation in human cancer genomes. *Nature* **578**, 112–121 (2020). 10.1038/s41586-019-1913-9 PMC7025897 32025012

[91] Rosenthal, R., McGranahan, N., Herrero, J., Taylor, B. S. & Swanton, C. DeconstructSigs: delineating mutational processes in single tumors distinguishes DNA repair deficiencies and patterns of carcinoma evolution. *Genome Biol.* **17**, 31 (2016). 26899170 10.1186/s13059-016-0893-4 PMC4762164

[92] Lawrence, M. et al. Software for computing and annotating genomic ranges. *PLoS Comput. Biol.* **9**, e1003118 (2013). 23950696 10.1371/journal.pcbi.1003118 PMC3738458

[93] Roberts, S. A. et al. An APOBEC cytidine deaminase mutagenesis pattern is widespread in human cancers. *Nat. Genet.* **45**, 970–976 (2013). 23852170 10.1038/ng.2702 PMC3789062

[94] Bailey, M. H. et al. Comprehensive characterization of cancer driver genes and mutations. *Cell* **173**, 371–385.e18 (2018). 29625053 10.1016/j.cell.2018.02.060 PMC6029450

[95] Karasaki, T. et al. Evolutionary characterization of lung adenocarcinoma morphology in TRACERx. *Nat. Med.*10.1038/s41591-023-02230-w (2023). 10.1038/s41591-023-02230-w PMC7614478 37045996

[96] Merino, D. M. et al. Establishing guidelines to harmonize tumor mutational burden (TMB): in silico assessment of variation in TMB quantification across diagnostic platforms: phase I of the Friends of Cancer Research TMB Harmonization Project. *J. Immunother. Cancer* **8**, e000147 (2020). 32217756 10.1136/jitc-2019-000147 PMC7174078

[97] Bentham, R. et al. Using DNA sequencing data to quantify T cell fraction and therapy response. *Nature* **597**, 555–560 (2021). 34497419 10.1038/s41586-021-03894-5

[98] Caswell, D. R. & Swanton, C. The role of tumour heterogeneity and clonal cooperativity in metastasis, immune evasion and clinical outcome. *BMC Med.* **15**, 133 (2017). 28716075 10.1186/s12916-017-0900-y PMC5514532

[99] Durinck, S., Spellman, P. T., Birney, E. & Huber, W. Mapping identifiers for the integration of genomic datasets with the R/Bioconductor package biomaRt. *Nat. Protoc.* **4**, 1184–1191 (2009). 19617889 10.1038/nprot.2009.97 PMC3159387

[100] Danaher, P. et al. Gene expression markers of tumor infiltrating leukocytes. *J. Immunother. Cancer* **5**, 18 (2017). 28239471 10.1186/s40425-017-0215-8 PMC5319024

[101] Han, L. et al. The genomic landscape and clinical relevance of A-to-I RNA editing in human cancers. *Cancer Cell* **28**, 515–528 (2015). 26439496 10.1016/j.ccell.2015.08.013 PMC4605878

[102] Zhang, M. et al. RNA editing derived epitopes function as cancer antigens to elicit immune responses. *Nat. Commun.* **9**, 3919 (2018). 30254248 10.1038/s41467-018-06405-9 PMC6156571

[103] Chen, L. et al. Recoding RNA editing of AZIN1 predisposes to hepatocellular carcinoma. *Nat. Med.* **19**, 209–216 (2013). 23291631 10.1038/nm.3043 PMC3783260

[104] Abadi, M. et al. TensorFlow: large-scale machine learning on heterogeneous distributed systems. Preprint at *arXiv*10.48550/arXiv.1603.04467 (2016).

[105] Lemaître, G., Nogueira, F. & Aridas, C. K. Imbalanced-learn: a Python toolbox to tackle the curse of imbalanced datasets in machine learning. *J. Mach. Learn. Res.* **18**, 559–563 (2017).

[106] McKinney, W. Data structures for statistical computing in python. In *Proc. 9th Python in Science Conf.* (eds van der Walt, S. & Millman, J.) 51–56 (SciPy, 2010).

[107] Pedregosa, F. et al. Scikit-learn: machine learning in Python. *J. Mach. Learn. Res.* **12**, 2825–2830 (2011).

