# Supplementary material for: Genomic–transcriptomic evolution in lung cancer and metastasis
Source: Nature. 2023 Apr 12;616(7957):543–52. doi: 10.1038/s41586-023-05706-4 (PMC10115639; doi:10.1038/s41586-023-05706-4)
Supplement: Supplementary file 1 — Supplementary file containing the CONSORT diagram detailing which samples from the TRACERx 421 whole-exome sequencing cohort were used for RNA-seq. [file 41586_2023_5706_MOESM1_ESM.pdf]

nature portfolio

https://doi.org/10.1038/s41586-023-05706-4

Supplementary information

Genomic–transcriptomic evolution in lung cancer and metastasis

In the format provided by the authors and unedited

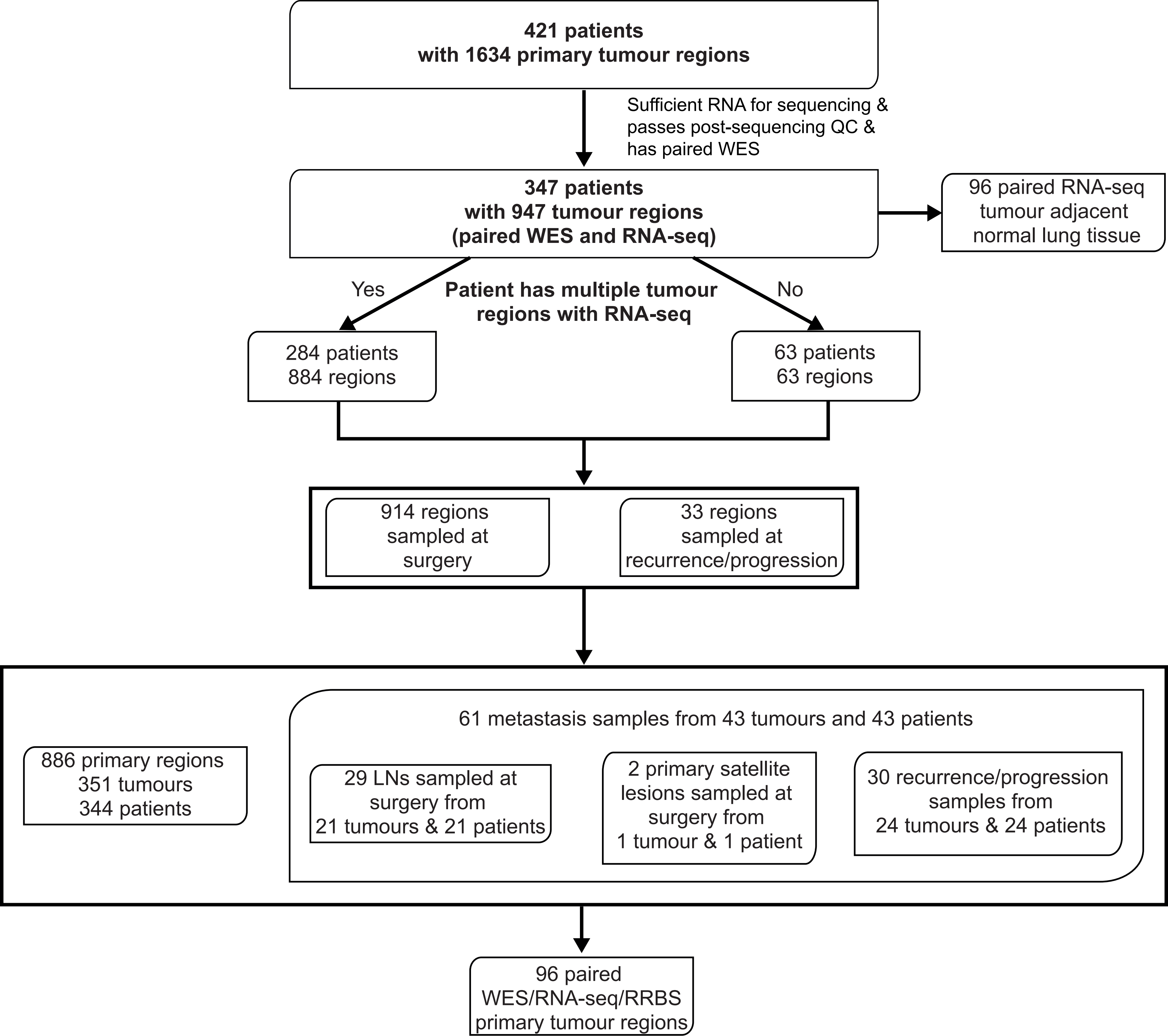
421 patients
with 1634 primary tumour regions
Sufficient RNA for sequencing &
passes post-sequencing QC &
has paired WES
347 patients
with 947 tumour regions
(paired WES and RNA-seq)
96 paired RNA-seq
tumour adjacent
normal lung tissue
Yes
Patient has multiple tumour
regions with RNA-seq
No
284 patients
884 regions
63 patients
63 regions
914 regions
sampled at
surgery
33 regions
sampled at
recurrence/progression
61 metastasis samples from 43 tumours and 43 patients
886 primary regions
351 tumours
344 patients
29 LNs sampled at
surgery from
21 tumours & 21 patients
2 primary satellite
lesions sampled at
surgery from
1 tumour & 1 patient
30 recurrence/progression
samples from
24 tumours & 24 patients
96 paired
WES/RNA-seq/RRBS
primary tumour regions
