# Supplementary material for: Genomic–transcriptomic evolution in lung cancer and metastasis
Source: Nature. 2023 Apr 12;616(7957):543–52. doi: 10.1038/s41586-023-05706-4 (PMC10115639; doi:10.1038/s41586-023-05706-4)
Supplement: Supplementary file 2 — Reporting Summary [file 41586_2023_5706_MOESM2_ESM.pdf]

nature portfolio

Corresponding author(s): Nicholas McGranahan
Charles Swanton

Last updated by author(s): Feb 3, 2023

Reporting Summary

Nature Portfolio wishes to improve the reproducibility of the work that we publish. This form provides structure for consistency and transparency in reporting. For further information on Nature Portfolio policies, see our Editorial Policies and the Editorial Policy Checklist.

Statistics

For all statistical analyses, confirm that the following items are present in the figure legend, table legend, main text, or Methods section.

n/a	Confirmed	
☐	☒	The exact sample size (*n*) for each experimental group/condition, given as a discrete number and unit of measurement
☐	☒	A statement on whether measurements were taken from distinct samples or whether the same sample was measured repeatedly
☐	☒	The statistical test(s) used AND whether they are one- or two-sided<br>*Only common tests should be described solely by name; describe more complex techniques in the Methods section.*
☐	☒	A description of all covariates tested
☐	☒	A description of any assumptions or corrections, such as tests of normality and adjustment for multiple comparisons
☐	☒	A full description of the statistical parameters including central tendency (e.g. means) or other basic estimates (e.g. regression coefficient) AND variation (e.g. standard deviation) or associated estimates of uncertainty (e.g. confidence intervals)
☐	☒	For null hypothesis testing, the test statistic (e.g. *F*, *t*, *r*) with confidence intervals, effect sizes, degrees of freedom and *P* value noted<br>*Give P values as exact values whenever suitable.*
☒	☐	For Bayesian analysis, information on the choice of priors and Markov chain Monte Carlo settings
☒	☐	For hierarchical and complex designs, identification of the appropriate level for tests and full reporting of outcomes
☐	☒	Estimates of effect sizes (e.g. Cohen's *d*, Pearson's *r*), indicating how they were calculated

Our web collection on statistics for biologists contains articles on many of the points above.

Software and code

Policy information about availability of computer code

Data collection

No software was used for data collection

Data analysis

RNA-seq alignment and QC
Illumina adapters were trimmed from raw sequencing reads using Cutadapt (v2.10)
The quality of the trimmed reads estimated per flow cell lane using FASTQC (v.0.11.9)
Fastq read files were aligned to the Hg19 human reference genome using STAR (v2.5.2a)
Duplicated reads in each BAM file were marked with the MarkDuplicates function from GATK (v4.1.7.0)
Aligned reads were quality checked using QoRTs (v1.3.6) to assess RNA integrity
Somalier (v0.2.7) was used to detect potential instances of sample mislabelling.
FASTQC, QoRTs and Somalier outputs were visualised using MultiQC (v1.9)
RSEM (v1.3.3) was used with default parameters to quantify gene expression based on the BAM files aligned to the transcriptome
RNA coverage was calculated for single nucleotide variants (SNVs) detected in matched whole exome sequencing data per tumour region using SAMtools (v1.9) mpileup
All steps described were implemented through the Nextflow (v20.07.1) pipeline manager

Reduced-representation Bisulfite Sequencing (RRBS)
FastQC v0.11.2 was used for quality control
Trim Galore! (Babraham Institute, https://www.babraham.ac.uk/) a wrapper around Cutadapt (v2.10), was used to trim reads
The bisulfite converted DNA sequence aligner Bismark (v0.14.4) was used to align reads to the UCSC reference genome Hg19
PCR deduplication was carried out using NuDup (v2.3), leveraging NuGEN's molecular tagging technology (https://github.com/nugentechnologies/nudup)

Most analyses were run using the R coding environment (v3.6.3)

RNA clustering
RSEM raw read counts were normalised using the median of ratios method implemented in DESeq2 (v1.24.0)
uniform manifold approximation and projection was performed using the R package umap (v2.7.7.0)
ASCAT (v2.3) and SAMTools mpileup (v1.9) were used to obtain RNA-derived estimates of tumour fraction

Gene expression differences
The R package edgeR (v3.26.5) was used to obtain gene expression differences
The R package fgsea (v1.10.1) was used to perform a gene set enrichment analysis on the gene expression differences results

Allele-specific expression
RNA read counts were compared to DNA copy number estimates through beta-binomial tests using the R package VGAM (v1.1-2)
CAMDAC (https://doi.org/10.1101/2020.11.03.366252) was used for allele-specific methylation calls

RNA variant calling
RNA-specific variants were called using the somatic variant caller Mutect2 and FilterMutectCalls from GATK (v4.1.7.0)
Mutect2 processes were run in parallel using GNU parallel (v20210422)
BCFtools (v1.10.2) was run to keep only biallelic PASS variants
bam-readcount (v0.8) was used to extract RNA reads at variant locations called by Mutect2 for further filtering, based on read depth and on the location of variants in the genome to prevent false positives arising from sequencing and mapping errors
RNA editing signatures were extracted using the R package hdp (v0.1.5)
Signatures were assigned to each tumour region using the R package deconstructSigs (v1.9.0)

All linear mixed effects models were performed using the R package lmerTest (v3.1-3)

The packages GenomicRanges (v1.36.0), stringr (v1.4.0) and TxDb.Hsapiens.UCSC.hg.knownGene (v3.2.2) were used to handle sequence data in R

dNdS analyses for detecting selection were performed usning the R package dndscv (v0.1.0.0)

The metastatic potential classifier was performed in Python (v3.3.5) using the packages pandas(v1.3.3), sklearn(v0.0) and tensorflow(v2.6.0)

The packages dplyr (v1.0.3), tidyr(v1.1.0) and reshape2 (v1.4.2) were used for data handling in R

Visualisation
Data was visualised using the R packages ggplot2 (v3.2.1), ggpubr (v0.4.0), cowplot (v1.0.0), gridExtra(v2.3), scales (v1.0.0) and ggrepel (v0.8.1)

For manuscripts utilizing custom algorithms or software that are central to the research but not yet described in published literature, software must be made available to editors and reviewers. We strongly encourage code deposition in a community repository (e.g. GitHub). See the Nature Portfolio guidelines for submitting code & software for further information.

Data

Policy information about availability of data

All manuscripts must include a data availability statement. This statement should provide the following information, where applicable:

- Accession codes, unique identifiers, or web links for publicly available datasets
- A description of any restrictions on data availability
- For clinical datasets or third party data, please ensure that the statement adheres to our policy

The RNA sequencing (RNA-seq), Whole exome sequencing (WES) and Reduced representation bisulfite sequencing (RRBS) data data (in each case from the TRACERx study) used during this study have been deposited at the European Genome–phenome Archive (EGA), which is hosted by The European Bioinformatics Institute (EBI) and the Centre for Genomic Regulation (CRG) under the accession codes EGAS00001006517 (RNAseq), EGAS00001006494 (WES) and EGAS00001006523 (RRBS); access is controlled by the TRACERx data access committee. Details on how to apply for access are available at the linked page.

Field-specific reporting

Please select the one below that is the best fit for your research. If you are not sure, read the appropriate sections before making your selection.

☒ Life sciences ☐ Behavioural & social sciences ☐ Ecological, evolutionary & environmental sciences

For a reference copy of the document with all sections, see nature.com/documents/nr-reporting-summary-flat.pdf

Life sciences study design

All studies must disclose on these points even when the disclosure is negative.

Sample size

The sample size (421 patients) represents the half-way point of the TRACERx longitudinal study. In total, we analysed paired whole exome sequencing and RNA-seq paired data from 347 patients that passed quality check filters for RNA.

TRACERx is a programme of work of multiple projects built around a single observational cohort study. It is not possible to perform a sample

size calculation for each project, especially post hoc. The study size of the cohort was done in relation to tumour heterogeneity and disease free survival:

The sample size is based on demonstrating a relationship between tumours with divergent intratumour heterogeneity index values and clinical outcome. Patients will be split evenly into those with a low and high intratumour heterogeneity index value (and other splits will be considered). Assuming a median Disease Free Survival (DFS) of 30 months and a hazard ratio (HR) of 0.77, with a 2-sided 5% significance level, 90% power, accrual period of 3 years and 5 years follow-up after the end of accrual, the sample size required is almost 400 per group (total of 800 patients). Assuming a 5% dropout rate, a total of 842 patients (421 per group) are required. At 85% power, 705 patients would be required in total, which could be the minimum target. However, we will instead aim for 750 patients and recruitment will continue for the length of time which is funded for accrual in order to get as close as possible to the ideal target of 842 patients. A study size of 842 is also large enough to detect a 10% improvement in a 5 year OS rate from 46% in the high Intratumour Heterogeneity Index (ITB) to 56% in the low Intratumour Heterogeneity Index group (HR=0.75), with 80% power and a 2 sided type I error set at 5% (logrank test). A high/low ITB value will be defined as values above/below the 50th percentile (median ITB). We have a target DFS effect of a 23% reduction in risk (hazard ratio 0.77), which means that our study is powered for an effect at least this large, including a 30% difference (which has been the target for progression-free survival in trials of advanced NSCLC, in relation to expected effects on OS).

Data exclusions

Data was excluded only on the basis of:
- Non-elegibility for the TRACERx clinical trial due to failure of the patient's data to comply with the study protocol (see below)
- The sequenced data did not pass our quality check filters

Replication

TRACERx is a prospective longitudinal study. As such, the results shown here are not the result of an experimental setup. This is the half-way point of the TRACERx 421 and reflects hypothesis generating analysis.

Randomization

Given the observational nature of the TRACERx longitudinal study, no experimental groups were allocated beforehand. Factors that could affect the interpretation of our results such as the background genetic makeup of each patient or the histological subtype of tumours were taken into account in all our statistical analyses. These were accounted for by including them as covariates in hypothesis testing. For instance, we used tumour ID as a random effect factor in linear mixed effects models for many of our analyses.

Blinding

Blinding is not relevant as this is an observational study. Patients were not allocated to any intervention and they were followed up and assessed as per routine practice. No biomarker results (tissue and bloods) are reported back to patients, so there is no likelihood of people changing their behaviours based on these findings. The laboratory analyses were all performed without knowing the outcome (DFS or survival) status of the patients, which represents a form of blinding.

Reporting for specific materials, systems and methods

We require information from authors about some types of materials, experimental systems and methods used in many studies. Here, indicate whether each material, system or method listed is relevant to your study. If you are not sure if a list item applies to your research, read the appropriate section before selecting a response.

Materials & experimental systems

n/a	Involved in the study
☒	☐ Antibodies
☒	☐ Eukaryotic cell lines
☒	☐ Palaeontology and archaeology
☒	☐ Animals and other organisms
☐	☒ Human research participants
☐	☒ Clinical data
☒	☐ Dual use research of concern

Methods

n/a	Involved in the study
☒	☐ ChIP-seq
☒	☐ Flow cytometry
☒	☐ MRI-based neuroimaging

Human research participants

Policy information about studies involving human research participants

Population characteristics

421 patients are included in this TRACERx cohort. 44.6% are females, 55.4% males; 93% are smokers or have a smoking history, 7% are never smokers; 25% of patients were diagnosed at stage IA, 25% at IB, 17.8% at IIA, 13.5% at IIB, 18.5% at IIIA and 0.2% and IIIB; 52% of diagnosed tumours were adenocarcinomas, 28.8% squamous cell carcinomas and 19.2, other histological subtypes; 93% of the cohort is from a white ethnic background and the mean age of the patients is 69, ranging between 34 and 92.

Please note that the study started recruiting patients in 2016, when TNM version 7 was standard of care. The up-to-date inclusion/exclusion criteria now utilizes TNM version 8.

TRACERx inclusion and exclusion criteria

Inclusion Criteria:
_Written Informed consent
_Patients ≥18 years of age, with early stage I-IIIB disease (according to TNM 8th edition) who are eligible for primary surgery.
_Histopathologically confirmed NSCLC, or a strong suspicion of cancer on lung imaging necessitating surgery (e.g. diagnosis determined from frozen section in theatre)
_Primary surgery in keeping with NICE guidelines planned

	_Agreement to be followed up at a TRACERx site<br>_Performance status 0 or 1<br>_Minimum tumor diameter at least 15mm to allow for sampling of at least two tumour regions (if 15mm, a high likelihood of nodal involvement on pre-operative imaging required to meet eligibility according to stage, i.e. T1N1-3)<br>Exclusion Criteria:<br>_Any other* malignancy diagnosed or relapsed at any time, which is currently being treated (including by hormonal therapy).<br>_Any other* current malignancy or malignancy diagnosed or relapsed within the past 3 years**.<br>*Exceptions are: non-melanomatous skin cancer, stage 0 melanoma in situ, and in situ cervical cancer<br>**An exception will be made for malignancies diagnosed or relapsed more than 2, but less than 3, years ago only if a pre-operative biopsy of the lung lesion has confirmed a diagnosis of NSCLC.<br>_Psychological condition that would preclude informed consent<br>_Treatment with neo-adjuvant therapy for current lung malignancy deemed necessary<br>_Post-surgery stage IV<br>_Known Human Immunodeficiency Virus (HIV), Hepatitis B Virus (HBV), Hepatitis C Virus (HCV) or syphilis infection.<br>_Sufficient tissue, i.e. a minimum of two tumor regions, is unlikely to be obtained for the study based on pre-operative imaging<br><br>Patient ineligibility following registration<br>_There is insufficient tissue<br>_The patient is unable to comply with protocol requirements<br>_There is a change in histology from NSCLC following surgery, or NSCLC is not confirmed during or after surgery.<br>_Change in staging to IIIC or IV following surgery<br>_The operative criteria are not met (e.g. incomplete resection with macroscopic residual tumors (R2)). Patients with microscopic residual tumors (R1) are eligible and should remain in the study<br>_Adjuvant therapy other than platinum-based chemotherapy and/or radiotherapy is administered.
Recruitment	When patients are initially diagnosed with stage I-III lung cancer and then referred for surgical resection, a research nurse identifies them on a clinic/operating list. The patient has an initial eligibility assessment and then provided with written information about the TRACERx study and he/she can ask the research nurse any questions.<br><br>Patients have to agree to provide serial blood samples whenever they attend clinic for routine blood sampling, so this represents the only main potential self-selecting bias (i.e. only patients willing to do this would participate). However, it is unclear how this would affect the biomarker analyses. Also, the gender and ethnicity characteristics are in line with patients seen in routine practice.<br><br>Inclusion and exclusion criteria are summarised above.<br>Informed consent for entry into the TRACERx study was mandatory and obtained from every patient.
Ethics oversight	The study was approved by the NRES Committee London with the following details:<br>Study title: TRAcking non small cell lung Cancer Evolution through therapy (Rx)<br>REC reference: 13/LO/1546<br>Protocol number: UCL/12/0279<br>IRAS project ID: 138871

Note that full information on the approval of the study protocol must also be provided in the manuscript.

Clinical data

Policy information about clinical studies

All manuscripts should comply with the ICMJE guidelines for publication of clinical research and a completed CONSORT checklist must be included with all submissions.

Clinical trial registration	TRACERx Lung https://clinicaltrials.gov/ct2/show/NCT01888601, approved by an independent Research Ethics Committee, 13/LO/1546
Study protocol	https://clinicaltrials.gov/ct2/show/NCT01888601
Data collection	Clinical and pathological data is collected from patients during study follow up - this period is a minimum of five years. Data collection is overseen by the sponsor of the study (Cancer Research UK & UCL Cancer Trials Centre) and takes place in hospitals across the United Kingdom. A centralised database called MACRO is used for this purpose. Recruitment started in 2014 across 6 sites (London, Leicester, Manchester, Aberdeen, Birmingham, and Cardiff) in the United Kingdom.
Outcomes	The main clinical outcomes are:<br>Disease-free survival (DFS) – measured from the time of study registration to date of first lung recurrence or death from any cause. Patients who do not have these events are censored at the date last known to be alive (including patients who developed a new primary tumour that has been shown biologically to not be linked to the initial primary lung tumour).<br>Overall survival - measured from the time of study registration to date of death from any cause.
